# Supplementary material for: Continuity of care, measurement and association with hospital admission and mortality: a registry-based longitudinal cohort study
Source: BMJ Open. 2021 Dec 1;11(12):e051958. doi: 10.1136/bmjopen-2021-051958 (PMC8640634; doi:10.1136/bmjopen-2021-051958)
Supplement: Supplementary data [file bmjopen-2021-051958supp001.pdf]

Supplemenary file A

Supplementary file A shows the estimated association between the three outcomes and the $UPC^{GP\ list}$ continuity index, using a piecewise linear function for age, income, number of GP visits, and number of hospital admissions (linear splines). All models were estimated with three knots at the 25th, 50th and 75th percentile for the continues variables.

Appendix table 1. Linear splines

	(1) Emergency admission	(2) Emergency admission for ACSC	(3) Mortality
Index score	-0.025***	-0.006*	0.000
	(-3.43)	(-1.99)	(0.35)
N	757873	757873	757873
R^2	0.092	0.051	0.050

t statistics in parentheses. Standard errors clustered at GP level.
In all estimations, control variables include individual patient characteristics listed in table 1 and municipality-fixed effects.
* $p < 0.05$, ** $p < 0.01$, *** $p < 0.001$

Hetlevik Ø, *et al. BMJ Open* 2022; 11:e051958. doi: 10.1136/bmjopen-2021-051958
