# Supplementary material for: Continuity of care, measurement and association with hospital admission and mortality: a registry-based longitudinal cohort study
Source: BMJ Open. 2021 Dec 1;11(12):e051958. doi: 10.1136/bmjopen-2021-051958 (PMC8640634; doi:10.1136/bmjopen-2021-051958)
Supplement: Author's manuscript [file bmjopen-2021-051958.draft_revisions.pdf]

BMJ Open is committed to open peer review. As part of this commitment we make the peer review history of every article we publish publicly available.

When an article is published we post the peer reviewers' comments and the authors' responses online. We also post the versions of the paper that were used during peer review. These are the versions that the peer review comments apply to.

The versions of the paper that follow are the versions that were submitted during the peer review process. They are not the versions of record or the final published versions. They should not be cited or distributed as the published version of this manuscript.

BMJ Open is an open access journal and the full, final, typeset and author-corrected version of record of the manuscript is available on our site with no access controls, subscription charges or pay-per-view fees (http://bmjopen.bmj.com).

If you have any questions on BMJ Open's open peer review process please email info.bmjopen@bmj.com

BMJ Open

Continuity of care, measurement and association with hospital admission and mortality – a registry-based longitudinal cohort study

Journal:	*BMJ Open*
Manuscript ID	bmjopen-2021-051958
Article Type:	Original research
Date Submitted by the Author:	01-Apr-2021
Complete List of Authors:	Hetlevik, Øystein; University of Bergen, Department of Global Public Health and Primary Care<br>Holmås, Tor ; NORCE Norwegian Research Centre AS Forskningsomrade Samfunn<br>Monstad, Karin; NORCE Norwegian Research Centre AS Forskningsomrade Samfunn
Keywords:	Organisation of health services < HEALTH SERVICES ADMINISTRATION & MANAGEMENT, PRIMARY CARE, Quality in health care < HEALTH SERVICES ADMINISTRATION & MANAGEMENT

SCHOLARONE™
Manuscripts

I, the Submitting Author has the right to grant and does grant on behalf of all authors of the Work (as defined in the below author licence), an exclusive licence and/or a non-exclusive licence for contributions from authors who are: i) UK Crown employees; ii) where BMJ has agreed a CC-BY licence shall apply, and/or iii) in accordance with the terms applicable for US Federal Government officers or employees acting as part of their official duties; on a worldwide, perpetual, irrevocable, royalty-free basis to BMJ Publishing Group Ltd ("BMJ") its licensees and where the relevant Journal is co-owned by BMJ to the co-owners of the Journal, to publish the Work in this journal and any other BMJ products and to exploit all rights, as set out in our licence.

The Submitting Author accepts and understands that any supply made under these terms is made by BMJ to the Submitting Author unless you are acting as an employee on behalf of your employer or a postgraduate student of an affiliated institution which is paying any applicable article publishing charge ("APC") for Open Access articles. Where the Submitting Author wishes to make the Work available on an Open Access basis (and intends to pay the relevant APC), the terms of reuse of such Open Access shall be governed by a Creative Commons licence – details of these licences and which Creative Commons licence will apply to this Work are set out in our licence referred to above.

Other than as permitted in any relevant BMJ Author's Self Archiving Policies, I confirm this Work has not been accepted for publication elsewhere, is not being considered for publication elsewhere and does not duplicate material already published. I confirm all authors consent to publication of this Work and authorise the granting of this licence.

For peer review only

Continuity of care, measurement and association with hospital admission and mortality – a registry-based longitudinal cohort study

Øystein Hetlevik, Associate Professor, Department of Global Public Health and Primary Care, University of Bergen, Bergen, Norway

Tor Helge Holmås, Research Professor, NORCE Norwegian Research Centre, Bergen, Norway

Karin Monstad, Research Professor, NORCE Norwegian Research Centre, Bergen, Norway

Corresponding author:
Øystein Hetlevik
E-mail: oystein.hetlevik@uib.no
Postal address:
Department of Global Public Health and Primary Care, University of Bergen,
Post-box 7804
N-5020 Bergen,
Norway

For peer review only

For peer review only - http://bmjopen.bmj.com/site/about/guidelines.xhtml

ABSTRACT

Objective To assess whether continuity of care (COC) with a general practitioner (GP) is associated with mortality and hospital admissions for older patients. We argue that the conventional continuity measure overestimates the associations, and present an alternative, service-based measure.

Design Registry-based, population-level longitudinal cohort study.

Setting Linked data from Norwegian administrative health care registries, including 3,989 GPs .

Participants 757,873 patients aged 60–90 years with ≥2 contacts with a GP during 2016 and 2017.

Main outcome measure All-cause emergency hospital admissions, emergency admissions for ambulatory care sensitive conditions, and mortality, in 2018.

Results We assessed continuity of care using the conventional usual provider of care index ($UPC^{patient}$) and an alternative/supplementary index ($UPC^{GP\ list}$) based on the COC for all other patients enlisted with the same preferred GP.

For both indices, the mean usual provider of care index score was 0.78. Our model controls for demographic and socio-economic characteristics, prior health care use, and municipality-fixed effects. Overall, $UPC^{GP\ list}$ shows a much weaker association between COC and the outcomes. For both indices, there is a negative relationship between COC and hospital admissions. A 0.2-point increase in the index score would reduce ambulatory care sensitive conditions by 8.1% (CI 7.1–9.1) versus merely 1.9% (0.2–3.5) according to $UPC^{patient}$ and $UPC^{GP\ list}$, respectively. Using $UPC^{GP\ list}$, we find that mortality is no longer associated with COC. There was greater evidence for an association between COC and all-cause admissions among patients with low education.

Conclusions

A continuity measure based on each patient's contacts with own preferred GP may overestimate the importance of COC as a feature of the GP practice. An alternative, service-based measure of continuity could be suitable as a quality measure in primary health care.

For peer review only - http://bmjopen.bmj.com/site/about/guidelines.xhtml

Facilitating continuity should be considered a health policy measure to reduce inequalities in health.

Strengths and limitations of this study

- This study is based on patient level data for the entire population aged 60-90 years, including all GP consultations and hospital admissions, and uses a longitudinal approach estimating continuity of care in a two-year period prior to the one-year period when outcomes are assessed.
- The study compares two continuity of care measures as predictors of patient outcomes: the conventional usual provider of care index and a service-based continuity of care index, measured at GP practice level.
- Continuity of care measured at GP practice level is associated with reduced number of acute hospital admissions, but not with mortality. The strength of the associations is markedly lower compared to the conventional individual measure.
- The conventional index may overestimate the relationship between continuity of care as a characteristic of GP care and the outcomes, because of unobserved individual health differences.
- A limitation is lack of detailed information of each patient's severity of disease, although we partly compensate for this by including prior health care use as a proxy for patients' health status.

For peer review only - http://bmjopen.bmj.com/site/about/guidelines.xhtml

Introduction

Continuity of care (COC) is a declared goal in primary care systems (1).The concept of COC is broad, but most often refers to personal continuity, either measured longitudinally or by standardised questionnaires eliciting information on patient-provider relational continuity (2-4). Additionally, the continuity concept includes informational continuity and management continuity as measures of level of seamless services across different care providers or sites (5). In this study, COC reflects the extent to which GP contacts are concentrated to the same GP in a given time period, which serves as a proxy for relational continuity. Recent literature reviews conclude that continuity is associated with lower mortality (6, 7) and a reduction in hospital admissions (8), indicating better patient treatment in primary care and lower total health care costs (9, 10). For chronic conditions that are considered manageable in primary care (ambulatory care sensitive conditions, ACSC), hospitalisations are considered a particularly sensitive marker of primary care quality (11, 12). Barker et al. (13) showed that longitudinal COC was associated with reduced number of hospital admissions for ACSCs. Despite the acknowledged virtues of continuity of care, there is a steadily increasing policy drive towards giving priority to swift access to care (14), which may contradict the possibility of seeing a preferred physician and challenge the goal of continuity of care (13, 15).

Since 2001, every inhabitant in Norway has been assigned a regular GP in a list patient system, and as such has a defined usual provider, while also having the option to switch GP twice in a calendar year. Improving continuity in the GP-patient relationship was one of the main arguments for this reform (16).

In this study, we contribute to the existing literature in several respects. First and foremost, we critically assess the conventional usual provider of care index, which we argue may

For peer review only - http://bmjopen.bmj.com/site/about/guidelines.xhtml

overestimate the association between emergency hospital admissions and COC as a feature of the GP service. The usual provider of care index represents, for each patient, the proportion of contacts with a preferred provider, most often a GP, in relation to all contacts during a given period (17). Our argument is that the conventional usual provider of care index disregards the fact that when the patient visits another GP than his/her preferred GP, he/she is in many cases in need of acute medical care, as discussed by Lustman et al. (18). Patients in acute need of health care are more inclined to be hospitalised, therefore patient unobserved health is a confounding factor that most likely leads to an overestimation of the (presumably negative) association between COC and hospital emergency admissions. Also, patients may prioritise differently between swift access and continuity of care, which may influence their relational COC independently of the COC offered at GP service level (19). We suggest an alternative measure, where COC is disentangled from each individual's unobserved health or personal choices. For each patient, we compute the continuity of care experienced by other patients having the same preferred GP. Our approach is detailed in the methods section.

Second, few studies have investigated how the importance of COC varies by patient characteristics (7), although COC seems to be of particular importance for patients in vulnerable groups (20). We measure the association between COC and outcomes for patients with different education levels. Third, we investigate how hospital admission and mortality are associated with different forms of discontinuity of care, where the patients instead of using the preferred GP either use another GP at ordinary opening hours or out-of-hours (OOH) services.

Since 95% of all Norwegian GPs work in group practices (21), patients may have relational continuity to several GPs who also share electronic health records. This facilitates informational and management continuity when the preferred GP is not available (22). In contrast, at OOH services, patients meet an unfamiliar physician with no access to the GP's

For peer review only - http://bmjopen.bmj.com/site/about/guidelines.xhtml

patient records, with low possibility for informational or management continuity to cover up for the broken relational continuity. It is not clear how informational and management continuity is associated with hospital admissions (8). Our data allow us to investigate how different kinds of discontinuity of care is associated with hospital admissions as well as mortality. Finally, to our knowledge, this is the first analysis of COC that benefits from patient and GP level data for the entire population.

For peer review only

Methods

Observational period

Several previous studies, e.g. Barker et al. (13), measure COC and the outcome within the same period. However, following Katz et al. (14), our empirical strategy is measuring continuity over a baseline period of two years (in our case, 2016–2017) before a follow-up period of one year (2018), when we measure the outcomes.

In this way, we avoid a potential reverse causality problem that arises because hospitalisation may lead to an increase in GP visits shortly after hospital discharge. If these visits are with own GP, COC for the relevant period will be measured as high, and the presumably negative association between COC and hospital admission will be washed out. Vice versa, if post-discharge visits are with another GP, the negative relationship between COC and hospitalisation will be overestimated.

In addition, when applying a baseline period separated from a follow-up period, we acknowledge that greater COC can lead to better disease control and delay or prevent the onset of end-stage disease.

For peer review only - http://bmjopen.bmj.com/site/about/guidelines.xhtml

Data sources

The analysis benefits from administrative registry data at the patient level, covering the entire population, with information on all GP visits in Norway (the KUHR registry). Data originate from reimbursement claims sent by the primary care physician following each consultation, whether at the GP's office or OOH visits at a primary care emergency centre. Since the KUHR registry includes patient identifiers and the consultation date, we can observe, for each patient, the number of primary physician visits during a given period. Furthermore, since the KUHR data also contain GP identifiers, they can be merged with information on each GP's patient list from the Regular GP database. This allows us to identify whether the visit was with the patient's own GP, another GP or at an OOH service.

Data on hospital admissions are provided by the Norwegian Patient Registry (NPR) and include information on all hospitalisations in Norway: the date of the admission, whether the admission was planned or unplanned, inpatient or outpatient, and diagnoses according to the ICD-10 system.

From Statistics Norway we obtained data on patient characteristics, including age, sex, education, income, whether the individual lives in a one-person household, and date of death.

Sample selection

Since COC is regarded especially important for patients with chronic conditions, which are more widespread in the elderly population, we restrict the sample to the age group 60–90. The number of inhabitants in this age group by January 1, 2016 is 1,100,010, of which nearly all, 1,099,268, are registered with a regular GP, while the group of regular GPs at this point counts 4,644. We apply the following sample selection criteria:

For peer review only - http://bmjopen.bmj.com/site/about/guidelines.xhtml

- the sample is restricted to patients who belong to the same GP-patient list in the baseline period. This criterion ensures that a unique preferred GP can be defined (in total 947,153 patients) and implies that we only include GPs practising by January 1, 2016 and throughout the baseline period (3,990 GPs).

- we exclude patients who die within the baseline period (53,366 patients).

- we restrict the sample to patients who have at least two consultations with primary care physicians during the baseline period (in total 757,873 patients and 3,989 GPs). This is in line with most previous studies using UPC as a measure of continuity (2, 13).

Outcomes

Our dependent variables are three-fold, and all outcomes are measured in 2018: (i) the number of *all-cause acute hospital admissions,* (ii) the number of *acute hospital admissions for ACSCs*, where the definition of ACSC is adapted from Bardsley et al. (23), also applied in Barker et al. (13), (iii) *mortality*, a variable that equals 1 if the individual dies during 2018.

Continuity of care measures

We apply two alternative definitions of the provider of care index, labelled $UPC^{patient}$ and $UPC^{GP\ list}$. Notably, in accordance with Norwegian health care policy, we define "the usual provider" as the regular GP that the patient is enlisted with. The $\text{UPC}^{\text{patient}}$ index is the traditional index applied in for instance Barker et al. (13). For instance, if an individual has 10 contacts in total, and 6 of them are with his own GP, the $\text{UPC}^{\text{patient}}$ score will be 0.6.

In order to disentangle the COC measure from individual *i'* s unobserved health and background characteristics (as explained in the introduction), we construct continuity measure

For peer review only - http://bmjopen.bmj.com/site/about/guidelines.xhtml

$UPC^{GP\ list}$. This index is based on the continuity of care experienced by *patients other than i* belonging to the same GP list. That implies that for each patient, we use information on the continuity experienced by other patients enlisted with *i*'s GP. An example could be a group of three patients A, B, C enlisted with the same GP. If A has 8 out of 10 visits with own GP, B has 3 out of 7, and C has 12 out of 12, then A's $UPC^{GP\ list}$ index score would be (3+12)/(7+12), B's score would be (8+12)/(10+12) and C's score would be (8+3)/(10+7). The $UPC^{GP\ list}$ index is in the interval (0,1), with a higher score reflecting greater continuity of care.

Covariates

To control for patient heterogeneity, we include information on a number of individual demographic and socio-economic characteristics: sex, age, level of education in three categories (primary, secondary and higher education), gross total income in NOK 100 000, and an indicator for one-person household (measured in 2016). Furthermore, utilisation of health care in the baseline period (2016–2017) may be indicative of the individual's health status. As controls, we therefore include the number of GP visits, the number of all-cause hospital admissions (whether planned or unplanned), the proportion of inpatient admissions, as well as the proportion unplanned hospital admissions.

Statistical analysis

We used multivariable regression with municipality-fixed effects (related to the municipality where the GP practice is located), to test the association between COC and the outcomes, number of hospital admissions and mortality. In these estimations, we included a series of covariates that may influence the association: individual demographic and socio-economic characteristics as well as health status indicators, as detailed above. Municipality-fixed effects

For peer review only - http://bmjopen.bmj.com/site/about/guidelines.xhtml

control for any time-invariant differences between municipalities, such as centrality, which may influence the availability of GPs – in particular, experienced GPs – as well as distance to hospital. Since our outcomes are either count data (number of admissions) or binary (mortality), non-linear models such as count data models or logit models may seem appropriate. However, estimating a fixed-effects logit model resulted in numerical overflow (due to too many effective observations). Therefore, we have chosen to estimate linear fixed effect models rather than non-linear models without fixed effect.

The relationship between COC and our outcomes is complex, and we do not claim that the current analysis detects causal pathways. For instance, it is conceivable that GPs offering greater COC attract a higher proportion of patients with chronic conditions, which would imply a selection bias. Still, we have tried to address the identification issues in several ways. First, we present an alternative index that we argue modifies the impact of patient unobserved health on observed COC. Second, information on a rich set of covariates helps reflect the individual's health status. Third, we observe these control variables in a baseline period *pre* outcomes. Finally, to improve inference, we take into account that patients enlisted with the same GP most likely share some (unobservable) characteristics, such as health and social background. To account for this heteroscedasticity, we cluster standard errors at the GP level in all estimations. Analyses were performed by STATA version 16.

Results

In total, 757,873 individuals, enlisted with 3,989 GPs, met our inclusion and exclusion criteria. In the following, we will compare results from using the conventional usual provider

For peer review only - http://bmjopen.bmj.com/site/about/guidelines.xhtml

of care index to results from using the service-based continuity of care measure presented above.

Figure 1 below reveals that for these two indices, mean scores are similar, yet there are large differences in the distribution. Note that for 22.5% of the sample, the $UPC^{patient}$ score equals one, i.e., for these patients, all their primary physician visits during the baseline period were with their own GP. Also, about 2.5 per cent of the sample had no visits to own GP, i.e., a $UPC^{patient}$ score of zero. By construction, the values zero and one do not appear when we apply the $UPC^{GP\ list}$ index.

*** INSERT FIGURE 1 HERE*****

Figure 1. Distribution of usual provider of care indices (*n*=757,873) based on data from individual patients (Left figure: $UPC^{patient}$) and at GP practice level (right figure: $UPC^{GP\ list}$). Red dotted lines: mean value of each index.

For peer review only

Table 1 reports summary statistics of outcome variables and covariates. We see that 53 per cent of the sample are women, the mean age is 70.7 years and 35 per cent live alone. The overall use of health care is high, about 9.4 GP visits and 5.1 hospital admissions during the two years' baseline period. This is to be expected, given the high age and the fact that a certain (low) level of GP use is an inclusion criterion. In the baseline period, the mean number of emergency hospital admissions per year is about 0.3 (11.9 per cent of 5.1 hospital admissions were emergency cases during these two years), while the mean number of all-cause hospital admissions in the follow-up year is slightly higher, 0.357. For the two COC measures, we see that the mean values are very similar (0.78), but the standard deviation is larger for the $UPC^{patient}$ index, as can also be seen from Figure 1.

Table 1. Descriptive statistics, patients aged 60–90 with at least two GP visits in 2016–2017. Proportions or means with standard errors in parentheses.

Outcomes (measured in 2018):	
No. of all-cause emergency hospital admissions	0.357 (0.939)
No. of admissions for ACSC[1]	0.068 (0.365)
Mortality	0.024
Continuity of care (measured at baseline, 2016–17):	
$UPC^{patient}$ [2]	0.788 (0.263)
$UPC^{GP\ list}$ [2]	0.780 (0.169)
Patient characteristics (measured at baseline, 2016–17):	
Proportion females	0.531
Age	70.675 (7.562)
Proportion low education[3]	0.259
Proportion medium education[3]	0.498
Proportion high education[3]	0.243
Total income[1] (100,000 NOK)	4.310 (5.067)
Proportion one-person household[3]	0.353
No. of GP visits	9.432 (7.967)
No. of hospital admissions	5.103 (9.884)
Proportion inpatient stays	0.093
Proportion emergency admissions	0.119
Number of patients	757,873

[1] ACSC: Ambulatory care sensitive conditions[2] $UPC^{patient}$ and $UPC^{GP\ list}$: Usual provider care index measured for each patient and for each GP practice, respectively.[3] Measured in 2016.

For peer review only - http://bmjopen.bmj.com/site/about/guidelines.xhtml

Table 2. Associations between continuity of care and the outcomes emergency admissions to hospital and mortality. Estimated with two different indices, standard errors clustered at GP level.

	Emergency admission		Emergency admission for ACSC[1]		Mortality	
	UPC^patient index[2]	UPC ^GP list index[2]	UPC ^patient index[2]	UPC ^GP list index[2]	UPC ^patient index[2]	UPC ^GP list index[2]
Continuity of care measure						
Index score	-0.104***	-0.028**	-0.027***	-0.006*	-0.010***	0.000
	(-22.16)	(-3.78)	(-15.67)	(-2.51)	(-13.42)	(0.28)
Patient characteristics						
Male	0.063***	0.063***	0.024***	0.024***	0.010***	0.009***
	(28.10)	(27.97)	(24.20)	(24.12)	(24.68)	(24.53)
Age	0.011***	0.011***	0.003***	0.003***	0.002***	0.002***
	(65.08)	(64.71)	(40.65)	(40.41)	(71.81)	(71.74)
Medium Education	-0.040***	-0.040***	-0.020***	-0.020***	-0.005***	-0.006***
	(-14.73)	(-14.88)	(-17.31)	(-17.40)	(-11.72)	(-11.80)
High Education	-0.050***	-0.050***	-0.031***	-0.031***	-0.008***	-0.008***
	(-15.65)	(-15.69)	(-22.12)	(-22.15)	(-15.75)	(-15.77)
Total income	-0.001*	-0.001*	-0.000	-0.000	-0.0001***	-0.0001***
	(-2.25)	(-2.10)	(-1.14)	(-1.09)	(-4.83)	(-4.62)
One-person household	0.040***	0.041***	0.011***	0.012***	0.007***	0.007***
	(17.51)	(17.89)	(12.52)	(12.79)	(18.55)	(18.82)
GP visits	0.017***	0.017***	0.005***	0.005***	0.0003***	0.0003***
	(65.63)	(65.31)	(42.85)	(42.72)	(7.39)	(7.24)
Hospital admissions	0.012***	0.012***	0.003***	0.003***	0.002***	0.002***
	(40.50)	(40.76)	(32.71)	(32.91)	(37.99)	(38.17)
Proportion inpatient	0.270***	0.272***	0.126***	0.126***	0.047***	0.047***
	(35.50)	(35.58)	(39.21)	(39.24)	(33.54)	(33.61)
Proportion unplanned	0.200***	0.213***	0.038***	0.042***	0.017***	0.019***
	(33.03)	(35.14)	(18.19)	(19.68)	(17.97)	(19.08)
R^2 (within)	0.088	0.088	0.049	0.049	0.046	0.043
Mean Y	0.357	0.357	0.068	0.068	0.024	0.024
Relative effect[3]	-5.8%	-1.6%	-8.1%	-1.9%	-8.3%	0.2%
N	757,873	757,873	757,873	757,873	757,873	757,873

t statistics in parentheses. * $p < 0.05$, ** $p < 0.01$, *** $p < 0.001$.
In addition to the covariates reported, estimations include municipality-fixed effects. [1] ACSC: Ambulatory care sensitive conditions. [2] UPC^patient and UPC^GP list : Usual provider care index measured for each patient and for each GP practice, respectively. [3] Percentage change in outcome when the index increases by 0.2.

Irrespective of COC measure (Table 2), we find a negative relationship between COC and emergency admissions, and notably, the estimated association is far stronger with the UPC^patient than with the UPC^GP list. Using the latter index, no relationship between continuity and mortality emerges. The following illustrates the difference in magnitude of estimates: when the index score increases by 0.2, the number of emergency admissions for ACSC is reduced by 8.1 per cent when measured with the UPC^patient and by merely 1.9 per cent when

For peer review only

For peer review only - http://bmjopen.bmj.com/site/about/guidelines.xhtml

measured with $UPC^{GP\ list}$. As expected, the relationship between COC and emergency admissions is stronger for ACSC than for other conditions, irrespective of index used.

The coefficient estimates of the control variables show the same pattern across outcomes: the number of emergency admissions as well as mortality is higher for males than for females, increasing in age, decreasing in education, virtually independent of income, and higher for individuals living in one-person households than for married/cohabitating. Individuals who are frequent users of GP or hospital visits in the baseline period have a higher number of hospital admissions and a higher mortality risk the following year.

Heterogeneity

In the following, we will investigate whether the relationship between COC (measured by the $UPC^{GP\ list}$ index) and our outcomes depends on patient characteristics. When we estimate separately by gender, we get qualitatively the same results as reported in Table 2, although with lower levels of significance. There is no clear difference in results between men and women (we have included an interaction term between the COC measure and gender, and its coefficient is not statistically significant, not reported here). Next, we estimate separately by the three levels of education. Regarding mortality, we find no statistically significant association for any of the subsamples defined by educational level. We therefore focus on the relationship between COC and hospital admission.

For peer review only - http://bmjopen.bmj.com/site/about/guidelines.xhtml

Table 3. Associations between continuity of care and emergency admissions to hospital, *by educational level*. Estimated with the UPC$^{GP\ list}$ index and standard errors clustered at GP level.

	Emergency admission			Emergency admission for ACSC[1]		
	Primary education	Secondary education	Higher education	Primary education	Secondary education	Higher education
Index score	-0.065***	-0.028**	0.010	-0.009	-0.007*	-0.000
	(-4.42)	(-2.73)	(0.78)	(-1.43)	(-2.09)	(-0.18)
R^2	0.093	0.086	0.075	0.011	0.046	0.038
Mean Y	0.440	0.342	0.298	0.099	0.063	0.043
Relative effect[2]	-2.6%	-1.3%	0.5%	-1.6%	-1.9%	-0.3%
N	196,482	377,635	183,756	196,482	377,635	183,756

t statistics in parentheses. * $p < 0.05$, ** $p < 0.01$, *** $p < 0.001$.
Control variables include individual patient characteristics and municipality-fixed effects (see Table 2). [1] ACSC: Ambulatory care sensitive conditions [2] Percentage change in outcome of a one standard deviation change in the index score.

From Table 3 we learn that the association between COC and all-cause emergency admissions is far stronger for patients with primary education than for the two other categories. There is no such socio-economic gradient for the outcome emergency admissions for ACSC.

Inspecting different forms of discontinuity of care

So far, our explanatory variable of interest has been the continuity with which patients on a GP list visit their own GP, while we have not distinguished between visits to other providers of primary doctor services. In our data, we can identify two categories of such consultations: visits to another GP at ordinary opening hours (a partner, registrar or locum), and OOH visits. As reported in Table 1, about 79% of all consultations are with own GP, while about 17% are with another GP, and 5% are OOH visits.

In the following, we will compare outcomes for individuals belonging to list populations that, to different degrees, visit other GPs or OOH services, respectively. We find this interesting to investigate, since different forms of broken COC bear different implications for informational and management continuity and probably also relational continuity. In this part of the

For peer review only - http://bmjopen.bmj.com/site/about/guidelines.xhtml

analysis, we therefore change our specification by replacing our COC measure with two new explanatory variables: the proportion of GP visits that is with another GP than the preferred GP, and the proportion of GP visits that is OOH visits (out of the total number of primary doctor visits in the GP list population). As before, we estimate outcomes at the individual level, controlling for municipality-fixed effects and individual patient characteristics related to the baseline period. Results are reported in Table 4.

For peer review only

Table 4. Associations between continuity of care and the outcomes emergency admissions to hospital and mortality. Estimated with the UPC$^{GP\ list}$ index and standard errors clustered at GP level.

	Emergency admission	Emergency admission for ACSC[1]	Mortality
Visits to other GPs, proportion at GP list level	0.014*	0.002	-0.000
	(1.99)	(0.71)	(-0.47)
Relative effect[2]	0.6%	0.5%	-0.2%
OOH visits, proportion at GP list level	0.959***	0.285***	0.017
	(9.57)	(7.45)	(1.49)
Relative effect[2]	5.3%	8.3%	1.4%
Mean y	0.357	0.068	0.02
R^2	0.088	0.048	0.046
Number of patients	757,873	757,873	757,873

t statistics in parentheses. * $p < 0.05$, ** $p < 0.01$, *** $p < 0.001$. Control variables include individual patient characteristics and municipality-fixed effects (see Table 2).
[1] ACSC: Ambulatory care sensitive conditions [2] Percentage change in outcome of a one standard deviation change in the index score.

We find that – for an individual patient in the list population – belonging to a list where OOH visits are more frequent is associated with a higher risk of both kinds of emergency admissions. On the other hand, emergency admissions for ASCS and mortality are both unrelated to the frequency (proportion) of consultations done by other GPs within their ordinary practice.

For peer review only - http://bmjopen.bmj.com/site/about/guidelines.xhtml

Discussion

Main findings

In this nationwide registry-based study using individual-level data from Norwegian health care, we investigated COC as predictor of acute hospital admissions and mortality among patients aged 60–90 years. We compared two measures of COC: the conventional usual provider of care index ($UPC^{patient}$) and an index defined for each GP list ($UPC^{GP\ list}$). Both indices were inversely associated with all-cause emergency hospital admissions and emergency admissions for ACSC. However, the associations between COC and these outcomes were considerably weaker when COC was measured with the index defined at GP level. We found no association between mortality and the $UPC^{GP\ list}$ index, while there was an estimated 8.3% decrease in mortality with a reduction of 0.2 in the conventional usual provider of care index.

Other things equal, emergency admissions are more common for individuals who belong to a GP list where the use of OOH visits is frequent. We found no or minimal increase in numbers of emergency admissions if patients, instead of using their preferred GP, visited another GP at the same GP practice.

Analysing subsamples defined by level of education, we found a clear gradient with the strongest association between COC and all-cause hospital admissions for patients with low education. No such gradient was found for ACSC admissions.

Interpretation of results

There is a policy drive towards reducing acute hospital admissions to reduce health care costs and streamline health services, and a well-functioning primary health care is judged to be important to achieve this (24). Earlier studies have found that COC is associated with a

For peer review only - http://bmjopen.bmj.com/site/about/guidelines.xhtml

reduction in all-cause acute hospital admissions (10, 14) as well as acute admissions for ACSCs (13), and our results support these findings. Recent studies report an inverse association between COC and mortality (7, 25). In our study, we found this result only when using the conventional usual provider of care index. The lack of (a negative) association between COC at practice level and mortality partly contradicts earlier findings (26, 27).

The literature assessing the impact of COC on secondary health care utilisation mainly applies data on the individual patient and his/her use of the preferred GP. In this paper, we add another perspective, by introducing an alternative index which reflects the COC in the preferred GP's practice population. Using this $UPC^{GP\ list}$ index, we found that the strength of COC as a predictor for admission attenuated markedly, and the $UPC^{GP\ list}$ index was not associated with mortality. These differences in associations between COC and the outcomes indicate that some patient characteristics are reflected in the conventional usual provider of care index that add to the effect of COC as a measure of health care performance. Even though we adjust for prior care contacts in our analyses, variation in patient unobserved health may influence the $UPC^{patient}$ index. A patient with more unstable disease is more often in need of swift access to care and might therefore more often visit other GPs or OOH services, and more often need a hospital admission, as also discussed by Lustman et al. (18). The result is a lower conventional usual provider of care index and a higher frequency of admissions, thereby increasing the COC effects, partly independent of the COC offered by the preferred GP during ordinary opening times. In addition, patients differ in their ability to manoeuvre in the health care system. Resourceful patients, who may also deal better with their diseases, may achieve a high COC even if the system level COC is low (19), and thereby also achieve the benefit of COC. This possibility is of course related to how the service is organised but will to a large extent reflect the patient's personal choices. A usual provider of care index

For peer review only - http://bmjopen.bmj.com/site/about/guidelines.xhtml

based on the individual patient's contacts with health care might overestimate the effect of COC as a feature of the GP practice, and a service-based measure of COC might be a more useful marker of care quality.

Our finding that the strength of the association between COC and all-cause acute hospital admissions is inversely related to educational level possibly reflects socio-economic differences in ways of using health care. An explanation could be that patients with lower education tend to be more dependent on the GP facilitating COC to help them achieve the benefit of better continuity (20), in line with the theory of advocacy set forward by Donaldson (28). Also, multi-morbidity is more common among patients with low education, and based on previous studies, COC is of greater importance under such conditions (29). However, we found no educational gradient with respect to acute ACSC hospital admissions, which could indicate a more equally distributed GP service related to ACSC, with standard guidelines and scheduled follow-up programs.

The present study shows that the number of emergency hospital admissions is almost unaffected by other GPs taking a larger share of consultations in the list population. On the other hand, there was a marked increase in frequency of hospital admissions if the $UPC^{GP\ list}$ was lower due to more frequent use of OOH services by patients in the list population. This indicates that the benefits of COC can be reaped also when using other GPs in a group practice, either because patients are familiar with more GPs ensuring relational continuity, or the lack of personal continuity can be compensated for by informational continuity (22). This finding supports a policy for increasing accessibility to GPs in daytime practices where a smaller group of GPs share responsibilities.

For peer review only - http://bmjopen.bmj.com/site/about/guidelines.xhtml

Strength and limitations

This analysis of COC is based on patient level data for the entire population, including all GP consultations and hospital admissions, combined with comprehensive data on patient characteristics and prior use of health care. In this analysis using claims data, health-related non-response is not a valid concern. Our findings are generalisable internationally in contexts where primary care is the first point of contact.

The longitudinal approach is also a strength, with COC estimation based on a two-year period prior to the one-year period when outcomes are assessed. A limitation is lack of information of each patient's severity of disease, although we partly compensate for this by including prior health care use as a proxy for health problems. A major weakness with registry data is that patient experiences with and evaluation of COC are not captured, whether at individual level or at GP list level. In the discussion about the connection between COC and hospital admissions, an underlying assumption is that reduced admission rates is a positive outcome, especially for ACSC where there is a special potential to stabilise patients with good routine care, and hospital admission rates are considered a performance indicator of primary health care. Still, based on data in the present study, we cannot know if the reduced frequency of hospital admissions benefits the individual patient, except we observe no increase in mortality.

In this analysis, we compare the conventional COC index to an alternative index. There are several indices in use, which are highly correlated (2, 13). We chose the conventional usual provider of care index as a starting point precisely because it is often used, the most straightforward to interpret and possible to adapt to estimation of COC delivered in a health care organisation, in this case a GP practice. A COC measure that is more directly measuring

For peer review only - http://bmjopen.bmj.com/site/about/guidelines.xhtml
For peer review only

performance of care, such as the proposed UPC^{GPlist} index, has a potential to be a useful quality measure, we argue. However, since our suggested index represents a new approach, it needs further assessment in research.

Implications and further research

The UPC$^{GP\ list}$ index is significantly associated with acute hospital admissions and could be a possible quality measure to inform policy decisions and follow up policy changes if the goal is to ensure COC in primary care. COC at practice level could be estimated using national registry data as in the present paper or by data available within a practice, as Sidaway-Lee et al. describe (30). However, whether a maximal gain of COC is achieved, seems to depend on patient characteristics in addition to health care performance. This is an important finding in relation to current changes in several health care systems, which emphasize quick access, private service providers and private insurance-based services for groups that may afford to buy these services, at risk of losing benefit of COC and probably increased total health care costs. Altogether, it seems that the health care system needs a primary care organisation that facilitates COC but also patients who are informed about the benefit of COC and willing to use services so they might achieve COC; but there is need for further research on how to promote COC. Continuity of care seems to be especially important for patients with low education, and prioritisation of COC by GPs and health care policy might be a contribution to reducing inequalities in health. A main goal for the list patient system is to provide equitable health care for all inhabitants, and variation in continuity of care and the related consequences shown is unwarranted variation that should be reduced.

For peer review only - http://bmjopen.bmj.com/site/about/guidelines.xhtml

GPs' personal responsibilities are extensive and work pressure is high. The expectation to deliver continuous care with high accessibility is increasingly problematic when recruiting and maintaining GPs (31, 32). Our findings indicate that GPs organised in group practices might achieve the same benefits of COC for their patients as a longitudinal, personal continuity with one regular GP, at least regarding the number of acute hospital admissions. This finding might support a stronger focus on common responsibilities between GPs in the future, but changes should be followed by more research on effects.

Footnotes

- Contributors: THH and KM outlined the study in the application for the funding grant. Together with ØH they further developed the project idea. THH had the main responsibility for analysing data, and all authors contributed to the interpretation of data. ØH and KM drafted the article, all authors reviewed and edited the manuscript, and approved the version that is submitted. The corresponding author attests that all listed authors meet authorship criteria and that no others meeting the criteria have been omitted.
- Funding: This study is part of a project funded by The Research Council of Norway, project number 288592
- Competing interests: All authors have completed the ICMJE uniform disclosure form at www.icmje.org/coi_disclosure.pdf and declare to have no conflict of interests
- Ethical approval: The study is approved by Committee for Medical and Health Research Ethics (Project number 2019/771/REKvest).
- Data sharing: Data are available from the national registries on a reasonable request. The use of data in this study is approved by application for this specific project only and data cannot be shared with anyone outside this project.

For peer review only - http://bmjopen.bmj.com/site/about/guidelines.xhtml

- The manuscript's guarantor (ØH) affirms that the manuscript is an honest, accurate, and transparent account of the study being reported; that no important aspects of the study have been omitted.
- Dissemination to participants and related patient and public communities: We are not able to disseminate the results of the research directly to study participants because the data used were anonymised, but plan to disseminate to community through general media general after the scientific publication.
- Patient and Public Involvement subsection: Patients or Public were not involved in the design or interpretation of this study
- Provenance and peer review: Not commissioned; externally peer reviewed.

For peer review only

For peer review only - http://bmjopen.bmj.com/site/about/guidelines.xhtml

References:

1. World Health O, United Nations Children's F. A vision for primary health care in the 21st century: towards universal health coverage and the Sustainable Development Goals. Geneva: World Health Organization; 2018.
2. Pollack CE, Hussey PS, Rudin RS, Fox DS, Lai J, Schneider EC. Measuring care continuity: a comparison of claims-based methods. Med Care. 2016;54(5):e30.
3. Saultz JW. Defining and Measuring Interpersonal Continuity of Care. Ann Fam Med. 2003;1(3):134-43.
4. Uijen AA, Schers HJ, van Weel C. Continuity of care preferably measured from the patients' perspective. J Clin Epidemiol. 2010;63(9):998-9.
5. Haggerty JL, Reid RJ, Freeman GK, Starfield BH, Adair CE, McKendry R. Continuity of care: a multidisciplinary review. BMJ. 2003;327(7425):1219-21.
6. Gray DJP, Sidaway-Lee K, White E, Thorne A, Evans PH. Continuity of care with doctors—a matter of life and death? A systematic review of continuity of care and mortality. BMJ open. 2018;8(6):e021161.
7. Baker R, Freeman GK, Haggerty JL, Bankart MJ, Nockels KH. Primary medical care continuity and patient mortality: a systematic review. Br J Gen Pract. 2020;70(698):e600-e11.
8. van Walraven C, Oake N, Jennings A, Forster AJ. The association between continuity of care and outcomes: a systematic and critical review. J Eval Clin Pract. 2010;16(5):947-56.
9. Nyweide DJ, Anthony DL, Bynum JPW, Strawderman RL, Weeks WB, Casalino LP, et al. Continuity of Care and the Risk of Preventable Hospitalization in Older Adults. JAMA Internal Medicine. 2013;173(20):1879-85.
10. Kohnke H, Zielinski A. Association between continuity of care in Swedish primary care and emergency services utilisation: a population-based cross-sectional study. Scand J Prim Health Care. 2017;35(2):113-9.
11. Freund T, Campbell SM, Geissler S, Kunz CU, Mahler C, Peters-Klimm F, et al. Strategies for reducing potentially avoidable hospitalizations for ambulatory care–sensitive conditions. Ann Fam Med. 2013;11(4):363-70.
12. Caminal J, Starfield B, Sánchez E, Casanova C, Morales M. The role of primary care in preventing ambulatory care sensitive conditions. Eur J Public Health. 2004;14(3):246-51.
13. Barker I, Steventon A, Deeny SR. Association between continuity of care in general practice and hospital admissions for ambulatory care sensitive conditions: cross sectional study of routinely collected, person level data. BMJ. 2017;356.
14. Katz DA, McCoy KD, Vaughan-Sarrazin MS. Does Greater Continuity of Veterans Administration Primary Care Reduce Emergency Department Visits and Hospitalization in Older Veterans? J Am Geriatr Soc. 2015;63(12):2510-8.
15. Bentler SE, Morgan RO, Virnig BA, Wolinsky FD. The association of longitudinal and interpersonal continuity of care with emergency department use, hospitalization, and mortality among Medicare beneficiaries. PLoS One. 2014;9(12):e115088-e.
16. Ministry of Health and Care Services. Regulation relating to a Municipal Regular GP Scheme. Oslo.2000 [Available from: http://www.regjeringen.no/en/dep/hod/dok/lover_regler/reglement/2000/regulation-relating-to-a-municipal-regul.html?id=420530.
17. Breslau N, Reeb KG. Continuity of care in a university-based practice. Acad Med. 1975;50(10):965-9.
18. Lustman A, Comaneshter D, Vinker S. Interpersonal continuity of care and type two diabetes. Prim Care Diabetes. 2016;10(3):165-70.

For peer review only - http://bmjopen.bmj.com/site/about/guidelines.xhtml

19. Boulton M, Tarrant C, Windridge K, Baker R, Freeman GK. How are different types of continuity achieved? A mixed methods longitudinal study. Br J Gen Pract. 2006;56(531):749-55.
20. Nutting PA, Goodwin MA, Flocke SA, Zyzanski SJ, Stange KC. Continuity of primary care: to whom does it matter and when? Ann Fam Med. 2003;1(3):149-55.
21. Norwegian Directorate of Health. Sammenstilling av data fra allmennlegetjenesten. Oslo.2019 [Available from: https://www.helsedirektoratet.no/rapporter/sammenstilling-av-data-fra-allmennlegetjenesten.
22. Forman JH, Robinson CH, Krein SL. Striving toward team-based continuity: provision of same-day access and continuity in academic primary care clinics. BMC Health Serv Res. 2019;19(1):145.
23. Bardsley M, Blunt I, Davies S, Dixon J. Is secondary preventive care improving? Observational study of 10-year trends in emergency admissions for conditions amenable to ambulatory care. BMJ Open. 2013;3(1).
24. Steventon A, Deeny S, Friebel R, Gardner T, Thorlby R. Briefing: emergency hospital admissions in England: which may be avoidable and how? The Health Foundation. 2018.
25. Pereira Gray DJ, Sidaway-Lee K, White E, Thorne A, Evans PH. Continuity of care with doctors—a matter of life and death? A systematic review of continuity of care and mortality. BMJ Open. 2018;8(6):e021161.
26. Honeyford K, Baker R, Bankart MJG, Jones D. Modelling factors in primary care quality improvement: a cross-sectional study of premature CHD mortality. BMJ Open 2013;3(10).
27. Levene LS, Bankart J, Khunti K, Baker R. Association of primary care characteristics with variations in mortality rates in England: an observational study. PLoS One. 2012;7(10):e47800.
28. Donaldson MS. Continuity of care: a reconceptualization. Med Care Res Rev. 2001;58(3):255-90.
29. Schers H, Webster S, van den Hoogen H, Avery A, Grol R, van den Bosch W. Continuity of care in general practice: a survey of patients' views. Br J Gen Pract. 2002;52(479):459-62.
30. Sidaway-Lee K, Gray DP, Evans P. A method for measuring continuity of care in day-to-day general practice: a quantitative analysis of appointment data. Br J Gen Pract. 2019;69(682):e356-e62.
31. Baird B, Charles A, Honeyman M, Maguire D, Das P. Understanding pressures in general practice. The King's Fund. 2016.
32. Ministry of Health and Care Services. Handlingsplan for allmennlegetjenesten. Attraktiv, kvalitetssikker og teambasert 2020-2024. Oslo.2020 [Available from: https://www.regjeringen.no/contentassets/96f6581333ee48559cdabf23c8772294/handlingsplan-for-allmennleger.pdf.

For peer review only - http://bmjopen.bmj.com/site/about/guidelines.xhtml

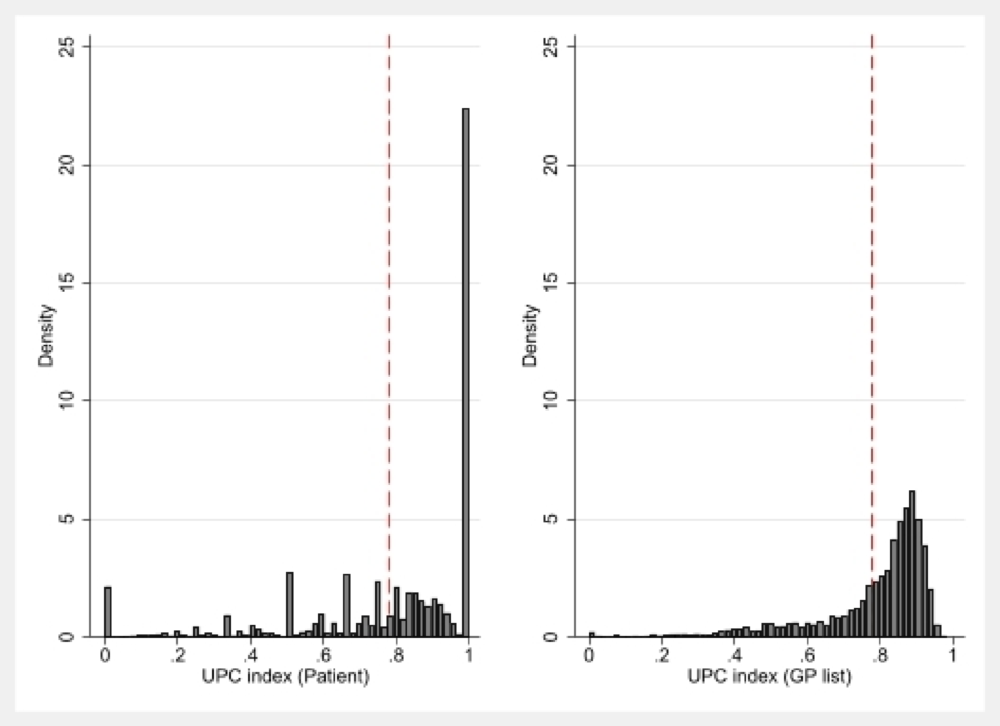

139x101mm (96 x 96 DPI)

The RECORD statement – checklist of items, extended from the STROBE statement, that should be reported in observational studies using routinely collected health data.

	Item No.	STROBE items	Location in manuscript where items are reported	RECORD items	Location in manuscript where items are reported
**Title and abstract**					
	1	(a) Indicate the study's design with a commonly used term in the title or the abstract (b) Provide in the abstract an informative and balanced summary of what was done and what was found		RECORD 1.1: The type of data used should be specified in the title or abstract. When possible, the name of the databases used should be included.<br><br>RECORD 1.2: If applicable, the geographic region and timeframe within which the study took place should be reported in the title or abstract.<br><br>RECORD 1.3: If linkage between databases was conducted for the study, this should be clearly stated in the title or abstract.	Abstract<br><br>Abstract<br><br>Abstract
**Introduction**					
Background rationale	2	Explain the scientific background and rationale for the investigation being reported			Background
Objectives	3	State specific objectives, including any prespecified hypotheses			Background page 5-6
**Methods**					
Study Design	4	Present key elements of study design early in the paper			Page 7-8
Setting	5	Describe the setting, locations, and relevant dates, including periods of recruitment, exposure, follow-up, and data collection			Page 7-8

For peer review only

Participants	6	*(a) Cohort study* - Give the eligibility criteria, and the sources and methods of selection of participants. Describe methods of follow-up<br>*Case-control study* - Give the eligibility criteria, and the sources and methods of case ascertainment and control selection. Give the rationale for the choice of cases and controls<br>*Cross-sectional study* - Give the eligibility criteria, and the sources and methods of selection of participants<br><br>*(b) Cohort study* - For matched studies, give matching criteria and number of exposed and unexposed<br>*Case-control study* - For matched studies, give matching criteria and the number of controls per case		RECORD 6.1: The methods of study population selection (such as codes or algorithms used to identify subjects) should be listed in detail. If this is not possible, an explanation should be provided.<br><br>RECORD 6.2: Any validation studies of the codes or algorithms used to select the population should be referenced. If validation was conducted for this study and not published elsewhere, detailed methods and results should be provided.<br><br>RECORD 6.3: If the study involved linkage of databases, consider use of a flow diagram or other graphical display to demonstrate the data linkage process, including the number of individuals with linked data at each stage.	Page 8-9 "sample selection"<br><br>Use complete population data, major selection is registered contacts, that is a valid variable in the national databases for health care use.<br><br>We have given a details description in page 8
Variables	7	Clearly define all outcomes, exposures, predictors, potential confounders, and effect modifiers. Give diagnostic criteria, if applicable.		RECORD 7.1: A complete list of codes and algorithms used to classify exposures, outcomes, confounders, and effect modifiers should be provided. If these cannot be reported, an explanation should be provided.	Described in sample section page 8 and following pages describing variables
Data sources/ measurement	8	For each variable of interest, give sources of data and details of methods of assessment (measurement).<br>Describe comparability of assessment methods if there is more than one group			Page 9-11

For peer review only

Bias	9	Describe any efforts to address potential sources of bias			
Study size	10	Explain how the study size was arrived at			Use complete population as source and database population
Quantitative variables	11	Explain how quantitative variables were handled in the analyses. If applicable, describe which groupings were chosen, and why			See "Variables" and "Statistical analyses" page 9-11
Statistical methods	12	(a) Describe all statistical methods, including those used to control for confounding<br>(b) Describe any methods used to examine subgroups and interactions<br>(c) Explain how missing data were addressed<br>(d) *Cohort study* - If applicable, explain how loss to follow-up was addressed<br>*Case-control study* - If applicable, explain how matching of cases and controls was addressed<br>*Cross-sectional study* - If applicable, describe analytical methods taking account of sampling strategy<br>(e) Describe any sensitivity analyses			Statistical methods are described page 10-11
Data access and cleaning methods		..		RECORD 12.1: Authors should describe the extent to which the investigators had access to the database population used to create the study population.	Use complete national register data as source, se page 7-8

For peer review only

				RECORD 12.2: Authors should provide information on the data cleaning methods used in the study.	Register use strict control on in-data. See also page7-8
Linkage		..		RECORD 12.3: State whether the study included person-level, institutional-level, or other data linkage across two or more databases. The methods of linkage and methods of linkage quality evaluation should be provided.	Linking described pages 7-8
**Results**					
Participants	13	(a) Report the numbers of individuals at each stage of the study (*e.g.*, numbers potentially eligible, examined for eligibility, confirmed eligible, included in the study, completing follow-up, and analysed)<br>(b) Give reasons for non-participation at each stage.<br>(c) Consider use of a flow diagram		RECORD 13.1: Describe in detail the selection of the persons included in the study (*i.e.,* study population selection) including filtering based on data quality, data availability and linkage. The selection of included persons can be described in the text and/or by means of the study flow diagram.	Page 11, selection procedures described in Methods
Descriptive data	14	(a) Give characteristics of study participants (*e.g.*, demographic, clinical, social) and information on exposures and potential confounders<br>(b) Indicate the number of participants with missing data for each variable of interest<br>(c) *Cohort study* - summarise follow-up time (*e.g.*, average and total amount)			Table 1
Outcome data	15	*Cohort study* - Report numbers of outcome events or summary measures over time			Table 2

For peer review only

		Case-control study - Report numbers in each exposure category, or summary measures of exposure<br>*Cross-sectional study* - Report numbers of outcome events or summary measures			
Main results	16	(a) Give unadjusted estimates and, if applicable, confounder-adjusted estimates and their precision (e.g., 95% confidence interval). Make clear which confounders were adjusted for and why they were included<br>(b) Report category boundaries when continuous variables were categorized<br>(c) If relevant, consider translating estimates of relative risk into absolute risk for a meaningful time period			Table 2 Unadjusted estimates are not given, to be able to present different models in the same tables.
Other analyses	17	Report other analyses done—e.g., analyses of subgroups and interactions, and sensitivity analyses			See "Statistical analyses" and table 3 and 4
**Discussion**					
Key results	18	Summarise key results with reference to study objectives			Page 18
Limitations	19	Discuss limitations of the study, taking into account sources of potential bias or imprecision. Discuss both direction and magnitude of any potential bias		RECORD 19.1: Discuss the implications of using data that were not created or collected to answer the specific research question(s). Include discussion of misclassification bias, unmeasured confounding, missing data, and changing eligibility over time, as they pertain to the study being reported.	Page 21

For peer review only

Interpretation	20	Give a cautious overall interpretation of results considering objectives, limitations, multiplicity of analyses, results from similar studies, and other relevant evidence			Page 22-23
Generalisability	21	Discuss the generalisability (external validity) of the study results			Page 21
**Other Information**					
Funding	22	Give the source of funding and the role of the funders for the present study and, if applicable, for the original study on which the present article is based			At the end of Methods
Accessibility of protocol, raw data, and programming code		..		RECORD 22.1: Authors should provide information on how to access any supplemental information such as the study protocol, raw data, or programming code.	The data are available from the national registers. Programming code be given by authors

*Reference: Benchimol EI, Smeeth L, Guttmann A, Harron K, Moher D, Petersen I, Sørensen HT, von Elm E, Langan SM, the RECORD Working Committee. The REporting of studies Conducted using Observational Routinely-collected health Data (RECORD) Statement. *PLoS Medicine* 2015; in press.

*Checklist is protected under Creative Commons Attribution (CC BY) license.

For peer review only

BMJ Open

Continuity of care, measurement and association with hospital admission and mortality – a registry-based longitudinal cohort study

Journal:	*BMJ Open*
Manuscript ID	bmjopen-2021-051958.R1
Article Type:	Original research
Date Submitted by the Author:	03-Sep-2021
Complete List of Authors:	Hetlevik, Øystein; University of Bergen, Department of Global Public Health and Primary Care<br>Holmås, Tor ; NORCE Norwegian Research Centre AS Research Area Social Sciences<br>Monstad, Karin; NORCE Norwegian Research Centre AS Research Area Social Sciences
**Primary Subject Heading**:	General practice / Family practice
Secondary Subject Heading:	Health services research
Keywords:	Organisation of health services < HEALTH SERVICES ADMINISTRATION & MANAGEMENT, PRIMARY CARE, Quality in health care < HEALTH SERVICES ADMINISTRATION & MANAGEMENT

SCHOLARONE™
Manuscripts

I, the Submitting Author has the right to grant and does grant on behalf of all authors of the Work (as defined in the below author licence), an exclusive licence and/or a non-exclusive licence for contributions from authors who are: i) UK Crown employees; ii) where BMJ has agreed a CC-BY licence shall apply, and/or iii) in accordance with the terms applicable for US Federal Government officers or employees acting as part of their official duties; on a worldwide, perpetual, irrevocable, royalty-free basis to BMJ Publishing Group Ltd ("BMJ") its licensees and where the relevant Journal is co-owned by BMJ to the co-owners of the Journal, to publish the Work in this journal and any other BMJ products and to exploit all rights, as set out in our licence.

The Submitting Author accepts and understands that any supply made under these terms is made by BMJ to the Submitting Author unless you are acting as an employee on behalf of your employer or a postgraduate student of an affiliated institution which is paying any applicable article publishing charge ("APC") for Open Access articles. Where the Submitting Author wishes to make the Work available on an Open Access basis (and intends to pay the relevant APC), the terms of reuse of such Open Access shall be governed by a Creative Commons licence – details of these licences and which Creative Commons licence will apply to this Work are set out in our licence referred to above.

Other than as permitted in any relevant BMJ Author's Self Archiving Policies, I confirm this Work has not been accepted for publication elsewhere, is not being considered for publication elsewhere and does not duplicate material already published. I confirm all authors consent to publication of this Work and authorise the granting of this licence.

For peer review only

Continuity of care, measurement and association with hospital admission and mortality – a registry-based longitudinal cohort study

Øystein Hetlevik, Professor, Department of Global Public Health and Primary Care, University of Bergen, Bergen, Norway

Tor Helge Holmås, Research Professor, NORCE Norwegian Research Centre, Bergen, Norway

Karin Monstad, Research Professor, NORCE Norwegian Research Centre, Bergen, Norway

Corresponding author:
Øystein Hetlevik
E-mail: oystein.hetlevik@uib.no
Postal address:
Department of Global Public Health and Primary Care, University of Bergen,
Post-box 7804
N-5020 Bergen,
Norway

For peer review only

For peer review only - http://bmjopen.bmj.com/site/about/guidelines.xhtml

ABSTRACT

Objective To assess whether continuity of care (COC) with a general practitioner (GP) is associated with mortality and hospital admissions for older patients We argue that the conventional continuity measure may overestimate these associations. To better reflect COC as a GP quality indicator, we present an alternative, service-based measure.

Design Registry-based, population-level longitudinal cohort study.

Setting Linked data from Norwegian administrative health care registries, including 3,989 GPs.

Participants 757,873 patients aged 60–90 years with ≥2 contacts with a GP during 2016 and 2017.

Main outcome measure All-cause emergency hospital admissions, emergency admissions for ambulatory care sensitive conditions, and mortality, in 2018.

Results We assessed continuity of care using the conventional usual provider of care index ($UPC^{patient}$) and an alternative/supplementary index ($UPC^{GP\ list}$) based on the COC for all other patients enlisted with the same preferred GP.

For both indices, the mean index score was 0.78. Our model controls for demographic and socio-economic characteristics, prior health care use, and municipality-fixed effects. Overall, $UPC^{GP\ list}$ shows a much weaker association between COC and the outcomes. For both indices, there is a negative relationship between COC and hospital admissions. A 0.2-point increase in the index score would reduce ambulatory care sensitive conditions by 8.1% (CI 7.1–9.1) versus merely 1.9% (0.2–3.5) according to $UPC^{patient}$ and $UPC^{GP\ list}$, respectively. Using $UPC^{GP\ list}$, we find that mortality is no longer associated with COC. There was greater evidence for an association between COC and all-cause admissions among patients with low education.

Conclusions

A continuity measure based on each patient's contacts with own preferred GP may overestimate the importance of COC as a feature of the GP practice. An alternative, service-based measure of continuity could be suitable as a quality measure in primary health care.

For peer review only - http://bmjopen.bmj.com/site/about/guidelines.xhtml

Facilitating continuity should be considered a health policy measure to reduce inequalities in health.

Strengths and limitations of this study

- This study is based on detailed nationwide registry data at the patient level for the entire population aged 60-90 years, including all GP consultations and hospital admissions.
- The analysis presents a novel service-based continuity of care index, measured at GP practice level, and discusses how this measure relates to the conventional usual provider of care index.
- We analyse how associations vary across gender and levels of education, and how different forms of breaches of COC are associated with patient outcomes.
- A limitation is lack of detailed information of each patient's severity of disease, although we partly compensate for this by including prior health care use as a proxy for patients' health status.
- Our data sources do not capture patient experiences with and evaluation of COC.

For peer review only

For peer review only - http://bmjopen.bmj.com/site/about/guidelines.xhtml

Introduction

Continuity of care (COC) is a declared goal in primary care systems (1). The concept of COC is broad, but most often refers to personal continuity, either measured longitudinally or by standardised questionnaires eliciting information on patient-provider relational continuity (2-4). Additionally, the continuity concept includes informational continuity and management continuity as measures of level of seamless services across different care providers or sites (5). In this study, COC reflects the extent to which GP contacts are concentrated to the same GP in a given time period, which serves as a proxy for relational continuity. Recent literature reviews conclude that continuity is associated with lower mortality (6, 7) and a reduction in hospital admissions (8), indicating better patient treatment in primary care and lower total health care costs (9, 10). For chronic conditions that are considered manageable in primary care (ambulatory care sensitive conditions, ACSC), hospitalisations are considered a particularly sensitive marker of primary care quality (11, 12). Barker et al. (13) showed that longitudinal COC was associated with reduced number of hospital admissions for ACSCs. Despite the acknowledged virtues of continuity of care, there is a steadily increasing policy drive towards giving priority to swift access to care (14), which may contradict the possibility of seeing a preferred physician and challenge the goal of continuity of care (13, 15).

Since 2001, more than 99% Norwegian inhabitants have been assigned a regular GP in a list patient system, and as such have a defined usual provider, while also having the option to switch GP twice in a calendar year. Improving continuity in the GP-patient relationship was one of the main arguments for this reform (16).

In this study, we contribute to the existing literature in several respects. First and foremost, we critically assess the conventional usual provider of care index, which we argue may overestimate the association between emergency hospital admissions and COC as a feature of

For peer review only - http://bmjopen.bmj.com/site/about/guidelines.xhtml

the GP service. The usual provider of care index represents, for each patient, the proportion of contacts with a preferred provider, most often a GP, in relation to all contacts during a given period (17). Our argument is that the conventional usual provider of care index disregards the fact that when the patient visits another GP than his/her preferred GP, he/she is in many cases in need of acute medical care, as discussed by Lustman et al. (18). Patients in acute need of health care are more inclined to be hospitalised, therefore patient unobserved health is a confounding factor that most likely leads to an overestimation of the (presumably negative) association between COC and hospital emergency admissions. Also, patients may prioritise differently between swift access to any GP and continuity of care with their regular GP, which may influence their relational COC independently of the COC offered at GP service level (19). We suggest an alternative measure, where COC is disentangled from each individual's unobserved health or personal choices. For each patient, we compute the continuity of care experienced by other patients having the same preferred GP. Our approach is detailed in the methods section.

Second, few studies have investigated how the importance of COC varies by patient characteristics (7), although COC seems to be of particular importance for patients in vulnerable groups (20). We measure the association between COC and outcomes for patients with different education levels. Third, we investigate how hospital admission and mortality are associated with different forms of discontinuity of care, where the patients instead of using the preferred GP either use another GP at ordinary opening hours or out-of-hours (OOH) services.

Since 95% of all Norwegian GPs work in group practices (21), patients may have relational continuity to several GPs who also share electronic health records. This facilitates informational and management continuity when the preferred GP is not available (22). In contrast, at OOH services, patients meet an unfamiliar physician with no access to the

For peer review only - http://bmjopen.bmj.com/site/about/guidelines.xhtml

preferred GP's patient records, with low possibility for informational or management continuity to cover up for the broken relational continuity. It is not clear how informational and management continuity is associated with hospital admissions (8). Our data allow us to investigate how different kinds of discontinuity of care is associated with hospital admissions as well as mortality. Finally, to our knowledge, this is the first analysis of COC that benefits from patient and GP level data for the entire population.

To sum up, our first aim is to assess whether COC with a GP is associated with mortality and hospital admissions for older patients. We focus on capturing how GP accessibility influences the association and present a GP service-based measure. Further, we aim to explore how the associations between the outcomes and the service-based COC measure vary across gender and educational level. Finally, we aim to analyse how these associations are influenced by breaches of personal COC compared with breaches of personal as well as informational COC.

Methods

Observational period

Several previous studies, e.g. Barker et al. (13), measure COC and the outcome within the same period. However, following Katz et al. (14), our empirical strategy is measuring continuity over a baseline period of two years (in our case, 2016–2017) before a follow-up period of one year (2018), when we measure the outcomes.

In this way, we avoid a potential reverse causality problem that arises because hospitalisation may lead to an increase in GP visits shortly after hospital discharge. If these visits are with own GP, COC for the relevant period will be measured as high, and the presumably negative association between COC and hospital admission will be washed out. Vice versa, if post-

For peer review only - http://bmjopen.bmj.com/site/about/guidelines.xhtml

discharge visits are with another GP, the negative relationship between COC and hospitalisation will be overestimated.

In addition, when applying a baseline period separated from a follow-up period, we acknowledge that greater COC can lead to better disease control and delay or prevent the onset of end-stage disease.

Data sources

The analysis benefits from administrative registry data at the patient level, covering the entire population, with information on all GP visits in Norway (the KUHR registry). Data originate from reimbursement claims sent by the primary care physician following each consultation, whether at the GP's office or OOH visits at a primary care emergency centre. Since the KUHR registry includes patient identifiers and the consultation date, we can observe, for each patient, the number of primary physician visits during a given period. Furthermore, since the KUHR data also contain GP identifiers, they can be merged with information on each GP's patient list from the Regular GP database. This allows us to identify whether the visit was with the patient's own GP, another GP or at an OOH service.

Data on hospital admissions are provided by the Norwegian Patient Registry (NPR) and include information on all hospitalisations in Norway: the date of the admission, whether the admission was planned or unplanned, inpatient or outpatient, and diagnoses according to the ICD-10 system.

From Statistics Norway we obtained data on patient characteristics, including age, sex, education, income, whether the individual lives in a one-person household, and date of death.

Sample selection

For peer review only - http://bmjopen.bmj.com/site/about/guidelines.xhtml

Since COC is regarded especially important for patients with chronic conditions, which are more widespread in the elderly population, we restrict the sample to the age group 60–90. The number of inhabitants in this age group by January 1, 2016 is 1,100,010, of which nearly all, 1,099,268, are registered with a regular GP, while the group of regular GPs at this point counts 4,644. We apply the following sample selection criteria:

- the sample is restricted to patients who belong to the same GP-patient list in the baseline period. This criterion ensures that a unique preferred GP can be defined (in total 947,153 patients) and implies that we only include GPs practising by January 1, 2016 and throughout the baseline period (3,990 GPs).
- we exclude patients who die within the baseline period (53,366 patients).
- we restrict the sample to patients who have at least two consultations with primary care physicians during the baseline period (in total 757,873 patients and 3,989 GPs). This is in line with most previous studies using UPC as a measure of continuity (2, 13).

Outcomes

Our dependent variables are three-fold, and all outcomes are measured in 2018: (i) the number of *all-cause acute hospital admissions,* (ii) the number of *acute hospital admissions for ACSCs*, where the definition of ACSC is adapted from Bardsley et al. (23), also applied in Barker et al. (13), (iii) *mortality*, a variable that equals 1 if the individual dies during 2018.

Continuity of care measures

We apply two alternative definitions of the provider of care index, labelled $UPC^{patient}$ and $UPC^{GP\ list}$. Notably, in accordance with Norwegian health care policy, we define "the usual provider" as the regular GP that the patient is enlisted with. The $UPC^{patient}$ index is the

For peer review only - http://bmjopen.bmj.com/site/about/guidelines.xhtml

traditional index applied in for instance Barker et al. (13). For instance, if an individual has 10 contacts in total, and 6 of them are with his own GP, the $UPC^{patient}$ score will be 0.6.

In order to disentangle the COC measure from individual *i'* s unobserved health and background characteristics (as explained in the introduction), we construct continuity measure $UPC^{GP\ list}$. This index is based on the continuity of care experienced by *patients other than i* belonging to the same GP list population. That implies that for each patient, we use information on the continuity experienced by other patients enlisted with *i*'s GP. An example could be a group of three patients A, B, C enlisted with the same GP. If A has 8 out of 10 visits with own GP, B has 3 out of 7, and C has 12 out of 12, then A's $UPC^{GP\ list}$ index score would be (3+12)/(7+12), B's score would be (8+12)/(10+12) and C's score would be (8+3)/(10+7). The UPC indices are in the interval (0,1), with a higher score reflecting greater continuity of care.

The $UPC^{GP\ list}$ index is a general concept, applicable irrespective of the size of the GP list population. If GP list populations are large, the $UPC^{GP\ list}$ index will be very similar to an index at GP level where all patients are included. We have estimated using such a simpler index, and results are virtually identical (not reported here).

When data availability allows it, the continuity of care analysis can include an investigation of the impact of different breaches of personal COC. For this purpose, we construct two indices of *dis*continuity: the proportion of all consultations in a GP list population that is with another GP at ordinary opening hours (Other-GP index) and the proportion of OOH visits (OOH - index), respectively.

Covariates

For peer review only - http://bmjopen.bmj.com/site/about/guidelines.xhtml

To control for patient heterogeneity, we include information on a number of individual demographic and socio-economic characteristics: sex, age, level of education in three categories (primary, secondary and higher education), gross total income in NOK 100 000, and an indicator for one-person household (measured in 2016). Furthermore, utilisation of health care in the baseline period (2016–2017) may be indicative of the individual's health status. As controls, we therefore include the number of GP visits, the number of all-cause hospital admissions (whether planned or unplanned), the proportion of inpatient admissions, as well as the proportion unplanned hospital admissions.

Statistical analysis

We used multivariable regression with municipality-fixed effects (related to the municipality where the GP practice is located), to test the association between COC and the outcomes, number of hospital admissions and mortality. In these estimations, we included a series of covariates that may influence the association: individual demographic and socio-economic characteristics as well as health status indicators, as detailed above. A fixed effects approach can reduce the impact of confounding by time-invariant factors (24). In the context of this analysis, municipality-fixed effects control for any time-invariant differences between municipalities, such as centrality, which may influence the availability of GPs – in particular, experienced GPs – as well as distance to hospital. Since our outcomes are either count data (number of admissions) or binary (mortality), non-linear models such as count data models or logit models may seem appropriate. However, estimating a fixed-effects logit model resulted in numerical overflow (due to too many effective observations). Therefore, we have chosen to estimate linear fixed effect models rather than non-linear models without fixed effect.

For peer review only - http://bmjopen.bmj.com/site/about/guidelines.xhtml

In stratified analyses, we estimate the associations separately by gender and by level of education. In an additional estimation, we use the two indices of *dis*continuity as main explanatory variables, while otherwise, the model specification is kept unchanged.

The non-categorical control variables are included in level form in all estimations reported in tables. As a sensitivity analysis, we have estimated linear splines models, which gave virtually identical results (for details, see Supplementary file A). The COC index enters the model linearly, which is common in the literature.

The relationship between COC and our outcomes is complex, and we do not claim that the current analysis detects causal pathways. For instance, it is conceivable that GPs offering greater COC attract a higher proportion of patients with chronic conditions, which would imply a selection bias. Still, we have tried to address the identification issues in several ways. First, we present an alternative index that we argue modifies the impact of patient unobserved health on observed COC. Second, information on a rich set of covariates helps reflect the individual's health status. Third, we observe these control variables in a baseline period *pre* outcomes. Finally, to improve inference, we take into account that patients enlisted with the same GP most likely share some (unobservable) characteristics that may influence outcomes, such as health, social background, and a preference for a certain GP practice style. These characteristics may not be fully adjusted for by municipality fixed effects, therefore, we cluster standard errors at the GP level.

Analyses were performed using STATA version 16.

Results

For peer review only - http://bmjopen.bmj.com/site/about/guidelines.xhtml

For peer review only

In total, 757,873 individuals, enlisted with 3,989 GPs, met our inclusion and exclusion criteria. In the following, we will compare results from using the conventional usual provider of care index to results from using the service-based continuity of care measure presented above.

Figure 1 below reveals that for these two indices, mean scores are similar, yet there are large differences in the distribution. Note that for 22.5% of the sample, the $UPC^{patient}$ score equals one, i.e., for these patients, all their primary physician visits during the baseline period were with their own GP. Also, about 2.5 per cent of the sample had no visits to own GP, i.e., a $UPC^{patient}$ score of zero. By construction, the values zero and one do not appear when we apply the $UPC^{GP\ list}$ index.

*** INSERT FIGURE 1 HERE*****

Figure 1. Distribution of usual provider of care indices (*n*=757,873) based on data from individual patients (Left figure: $UPC^{patient}$) and at GP practice level (right figure: $UPC^{GP\ list}$). Red dotted lines: mean value of each index.

For peer review only - http://bmjopen.bmj.com/site/about/guidelines.xhtml

For peer review only

Table 1 reports summary statistics of outcome variables and covariates. We see that 53 per cent of the sample are women, the mean age is 70.7 years and 35 per cent live alone. The overall use of health care is high, about 9.4 GP visits and 5.1 hospital admissions during the two years' baseline period. This is to be expected, given the high age and the fact that a certain (low) level of GP use is an inclusion criterion. In the baseline period, the mean number of emergency hospital admissions per year is about 0.3 (11.9 per cent of 5.1 hospital admissions were emergency cases during these two years), while the mean number of all-cause hospital admissions in the follow-up year is slightly higher, 0.357. For the two COC measures, we see that the mean values are very similar (0.78), but the standard deviation is larger for the UPC^{patient} index, as can also be seen from Figure 1.

Table 1. Descriptive statistics, patients aged 60–90 with at least two GP visits in 2016–2017. Proportions or means with standard errors in parentheses.

Continuity of care (measured at baseline, 2016–17):	
UPC$^{patient\ 2}$	0.788 (0.263)
UPC$^{GP\ list\ 2}$	0.780 (0.169)
Patient characteristics (measured at baseline, 2016–17):	
Proportion females	0.531
Age	70.675 (7.562)
Proportion low education[3]	0.259
Proportion medium education[3]	0.498
Proportion high education[3]	0.243
Total income[1] (100,000 NOK)	4.310 (5.067)
Proportion one-person household[3]	0.353
No. of GP visits	9.432 (7.967)
No. of hospital admissions	5.103 (9.884)
Proportion inpatient stays	0.093
Proportion emergency admissions	0.119
Number of patients	757,873

[1] ACSC: Ambulatory care sensitive conditions[2] UPC^{patient} and UPC$^{GP\ list}$: Usual provider care index measured for each patient and for each GP practice, respectively.[3] Measured in 2016.

Table 2. Associations between continuity of care and the outcomes emergency admissions to hospital and mortality. Estimated with two different indices, standard errors clustered at GP level.

	Emergency admission		Emergency admission for ACSC[1]		Mortality	
	UPC^{patient} index[2]	UPC $^{GP list}$ index[2]	UPC ^{patient} index[2]	UPC $^{GP list}$ index[2]	UPC ^{patient} index[2]	UPC $^{GP list}$ index[2]
Index score	-0.104***	-0.028**	-0.027***	-0.006*	-0.010***	0.000
	(-22.16)	(-3.78)	(-15.67)	(-2.51)	(-13.42)	(0.28)
R^2 (within)	0.088	0.088	0.049	0.049	0.046	0.043
Mean Y	0.357	0.357	0.068	0.068	0.024	0.024
Relative effect[3]	-5.8%	-1.6%	-8.1%	-1.9%	-8.3%	0.2%
N	757,873	757,873	757,873	757,873	757,873	757,873

t statistics in parentheses. * $p < 0.05$ ** $p < 0.01$, *** $p < 0.001$.
In all estimations, control variables include individual patient characteristics listed in table 1 and municipality-fixed effects. [1]ACSC: Ambulatory care sensitive conditions. [2]UPC^{patient} and UPC$^{GP list}$: Usual provider care index measured for each patient and for each GP practice, respectively. [3]Percentage change in outcome when the index increases by 0.2.

Irrespective of COC measure (Table 2), we find a negative relationship between COC and emergency admissions, and notably, the estimated association is far stronger with the UPC^{patient} than with the UPC$^{GP list}$. Using the latter index, no relationship between continuity and mortality emerges. The following illustrates the difference in magnitude of estimates: when the index score increases by 0.2, the number of emergency admissions for ACSC is reduced by 8.1 per cent when measured with the UPC^{patient} and by merely 1.9 per cent when measured with UPC$^{GP list}$. As expected, the relationship between COC and emergency admissions is stronger for ACSC than for other conditions, irrespective of index used.

The coefficient estimates of the control variables show the same pattern across outcomes: the number of emergency admissions as well as mortality is higher for males than for females, increasing in age, decreasing in education, virtually independent of income, and higher for individuals living in one-person households than for married/cohabitating. Individuals who are frequent users of GP or hospital visits in the baseline period have a higher number of hospital admissions and a higher mortality risk the following year.

Heterogeneity

For peer review only - http://bmjopen.bmj.com/site/about/guidelines.xhtml

In the following, we will investigate whether the relationship between COC (measured by the UPC$^{GP\ list}$ index) and our outcomes depends on patient characteristics. When we estimate separately by gender, we get qualitatively the same results as reported in Table 2, although with lower levels of significance. There is no clear difference in results between men and women (we have included an interaction term between the COC measure and gender, and its coefficient is not statistically significant, not reported here). Next, we estimate separately by the three levels of education. Regarding mortality, we find no statistically significant association for any of the subsamples defined by educational level. We therefore focus on the relationship between COC and hospital admission.

Table 3. Associations between continuity of care and emergency admissions to hospital, stratified analyses by educational level. Estimated with the UPC$^{GP\ list}$ index and standard errors clustered at GP level.

	Emergency admission			Emergency admission for ACSC[1]		
	Primary education	Secondary education	Higher education	Primary education	Secondary education	Higher education
Index score	-0.065***	-0.028**	0.010	-0.009	-0.007*	-0.000
	(-4.42)	(-2.73)	(0.78)	(-1.43)	(-2.09)	(-0.18)
R^2	0.093	0.086	0.075	0.011	0.046	0.038
Mean Y	0.440	0.342	0.298	0.099	0.063	0.043
Relative effect[2]	-2.6%	-1.3%	0.5%	-1.6%	-1.9%	-0.3%
N	196,482	377,635	183,756	196,482	377,635	183,756

t statistics in parentheses. * $p < 0.05$, ** $p < 0.01$, *** $p < 0.001$.
Control variables include individual patient characteristics listed in table 1and municipality-fixed effects.
[1]ACSC: Ambulatory care sensitive conditions. [2]Percentage change in outcome of a one standard deviation change in the index score.

From Table 3 we learn that the association between COC and all-cause emergency admissions is far stronger for patients with primary education than for the two other categories. There is no such socio-economic gradient for the outcome emergency admissions for ACSC.

Inspecting different forms of discontinuity of care

So far, our explanatory variable of interest has been the continuity with which patients in a GP list population visit their own GP, while we have not distinguished between visits to other

For peer review only - http://bmjopen.bmj.com/site/about/guidelines.xhtml

providers of primary doctor services. We now change the focus of the analysis, to investigate the relationship between the outcomes studied and different forms of *dis*continuity of care. In our data, we can identify two categories of such consultations: visits to another GP at ordinary opening hours (a partner, registrar or locum), and OOH visits. As reported in Table 1, about 79% of all consultations are with own GP, while about 17% are with another GP, and 5% are OOH visits.

In the following, we will compare outcomes for individuals belonging to list populations that, to different degrees, visit other GPs or OOH services, respectively. We find this interesting to investigate, since different forms of broken COC bear different implications for informational and management continuity and probably also relational continuity. In this part of the analysis, we therefore change our specification by replacing our COC measure with two new explanatory variables: (i) Other-GP index (the proportion of GP visits that is with another GP than the preferred GP during ordinary opening hours), and (ii) the OOH-index (the proportion of GP visits that is OOH visits). Both proportions are out of the total number of GP and OOH doctor visits in the GP list population.

As before, we estimate outcomes at the individual level, controlling for municipality-fixed effects and individual patient characteristics related to the baseline period. Results are reported in Table 4.

Table 4. Associations between proportion of consultations with other GPs or OOH services in list population of the patient's regular GP and the emergency admissions to hospital and mortality.

For peer review only - http://bmjopen.bmj.com/site/about/guidelines.xhtml

	Emergency admission	Emergency admission for ACSC[1]	Mortality
Other-GP index	0.014^{*}	0.002	-0.000
	(1.99)	(0.71)	(-0.47)
OOH-index	0.959^{***}	0.285^{***}	0.017
	(9.57)	(7.45)	(1.49)
R^2	0.088	0.048	0.046
Mean Y	0.357	0.068	0.024
Relative effect variable “Other GP index”[2]	0.6%	0.5%	-0.2%
Relative effect variable «OOH-index» [2]	5.3%	8.3%	1.4%
N	757,873	757,873	757,873

t statistics in parentheses. * $p < 0.05$, ** $p < 0.01$, *** $p < 0.001$. Control variables include individual patient characteristics listed in table 1 and municipality-fixed effects. Standard errors clustered at GP level
[1]ACSC: Ambulatory care sensitive conditions. [2]Percentage change in outcome of a one standard deviation change in the index score.

We find that – for an individual patient in the list population – belonging to a list where OOH visits are more frequent is associated with a higher risk of both kinds of emergency admissions. On the other hand, emergency admissions for ASCS and mortality are both unrelated to the frequency (proportion) of consultations done by other GPs within their ordinary practice. We also find that GPs rarely treats patients belonging to a list outside their group practice since such cases qualify for a particular fee, triggered in merely 0.6% of consultations.

Discussion

Main findings

In this nationwide registry-based study using individual-level data from Norwegian health care, we investigated COC as predictor of acute hospital admissions and mortality among patients aged 60–90 years. We compared two measures of COC: the conventional usual provider of care index ($UPC^{patient}$) and an index defined for each GP list population ($UPC^{GP\ list}$). In line with earlier studies, we find a marked association between $UPC^{patient}$ and lower rates for acute admissions and mortality. Both indices were inversely associated with all-cause emergency hospital admissions and emergency admissions for ACSC. However, the

For peer review only - http://bmjopen.bmj.com/site/about/guidelines.xhtml

associations between COC and these outcomes were considerably weaker when COC was measured with the index defined at GP level. We found no association between mortality and the $UPC^{GP\ list}$ index, while there was an estimated 8.3% decrease in mortality with a reduction of 0.2 in the conventional usual provider of care index.

Analysing subsamples defined by level of education, we found a clear gradient with the strongest association between COC and all-cause hospital admissions for patients with low education. No such gradient was found for ACSC admissions.

Other things equal, emergency admissions are more common for individuals who belong to a GP list population where the use of OOH visits is frequent. We found no or minimal increase in number of emergency admissions if patients, instead of using their preferred GP, visited another GP. Data tell us that the "other GP" very rarely treats patients from outside the patient lists of his/her group practice. Therefore, we infer that, as a rule, "the other GP" and the preferred GP work within the same GP practice.

Interpretation of results

There is a policy drive towards reducing acute hospital admissions to reduce health care costs and streamline health services, and a well-functioning primary health care is judged to be important to achieve this (25). Earlier studies have found that COC is associated with a reduction in all-cause acute hospital admissions (10, 14) as well as acute admissions for ACSCs (13), and our results support these findings. Recent studies report an inverse association between COC and mortality (7, 26). In our study, we found this result only when using the conventional usual provider of care index. The lack of (a negative) association between COC at practice level and mortality partly contradicts earlier findings (27, 28).

For peer review only - http://bmjopen.bmj.com/site/about/guidelines.xhtml

The literature assessing the impact of COC on secondary health care utilisation mainly applies data on the individual patient and his/her use of the preferred GP. Our study supports the positive association between such measures of COC and positive health outcomes. However, in this paper, we add another perspective, by introducing an alternative index which reflects the COC in the preferred GP's practice population. Using this $UPC^{GP\ list}$ index, we found that the strength of COC as a predictor for admission attenuated markedly, and the $UPC^{GP\ list}$ index was not associated with mortality. These differences in associations between COC and the outcomes indicate that some patient characteristics or choices are reflected in the conventional usual provider of care index that add to the effect of COC as a measure of health care performance. Even though we adjust for prior care contacts in our analyses, variation in patient unobserved health may influence the $UPC^{patient}$ index. A patient with more unstable disease is more often in need of swift access to care and might therefore more often visit other GPs or OOH services, and more often need a hospital admission, as also discussed by Lustman et al. (18). The result is a lower conventional usual provider of care index and a higher frequency of admissions, thereby increasing the COC effects, partly independent of the COC offered by the preferred GP during ordinary opening times. In addition, patients differ in their ability to manoeuvre in the health care system. Resourceful patients, who may also deal better with their diseases, may achieve a high COC even if the system level COC is low (19), and thereby also achieve the benefit of COC. This possibility is of course related to how the service is organised but will to a large extent reflect the patient's personal choices. A usual provider of care index based on the individual patient's contacts with health care might overestimate the effect of COC as a feature of the GP practice, and a service-based measure of COC might be a more useful marker of care quality.

For peer review only - http://bmjopen.bmj.com/site/about/guidelines.xhtml

Our finding that the strength of the association between COC and all-cause acute hospital admissions is inversely related to educational level possibly reflects socio-economic differences in ways of using health care. An explanation could be that patients with lower education tend to be more dependent on the GP facilitating COC to help them achieve the benefit of better continuity (20), in line with the theory of advocacy set forward by Donaldson (29). Also, multi-morbidity is more common among patients with low education, and based on previous studies, COC is of greater importance under such conditions (30). However, we found no educational gradient with respect to acute ACSC hospital admissions, which could indicate a more equally distributed GP service related to ACSC, with standard guidelines and scheduled follow-up programs.

The present study shows that the number of emergency hospital admissions is almost unaffected by other GPs taking a larger share of consultations in the list population. On the other hand, there was a marked increase in frequency of hospital admissions if the $UPC^{GP\ list}$ was lower due to more frequent use of OOH services by patients in the list population. This indicates that the benefits of COC can be reaped also when using other GPs in a group practice, either because patients are familiar with more GPs ensuring relational continuity, or the lack of personal continuity can be compensated for by informational continuity (22). This finding supports a policy for increasing accessibility to GPs in daytime practices where a smaller group of GPs share responsibilities.

Strength and limitations

This analysis of COC is based on patient level data for the entire population, including all GP consultations and hospital admissions, combined with comprehensive data on patient characteristics and prior use of health care. In this analysis using claims data, health-related

For peer review only - http://bmjopen.bmj.com/site/about/guidelines.xhtml

non-response is not a valid concern. Our findings are generalisable internationally in contexts where primary care is the first point of contact.

The longitudinal approach is also a strength, with COC estimation based on a two-year period prior to the one-year period when outcomes are assessed. A limitation is lack of information of each patient's severity of disease, although we partly compensate for this by including prior health care use as a proxy for health problems. A major weakness with registry data is that patient experiences with and evaluation of COC are not captured, whether at individual level or at GP list population level. In the discussion about the connection between COC and hospital admissions, an underlying assumption is that reduced admission rates is a positive outcome, especially for ACSC where there is a special potential to stabilise patients with good routine care, and hospital admission rates are considered a performance indicator of primary health care. Still, based on data in the present study, we cannot know if the reduced frequency of hospital admissions benefits the individual patient, except we observe no increase in mortality.

In this analysis, we compare the conventional COC index to an alternative index. There are several indices in use, which are highly correlated (2, 13). We chose the conventional usual provider of care index as a starting point precisely because it is often used, the most straightforward to interpret and possible to adapt to estimation of COC delivered in a health care organisation, in this case a GP practice. A COC measure that is more directly measuring performance of care, such as the proposed UPC^{GPlist} index, has a potential to be a useful quality measure, we argue.

Implications and further research

For peer review only - http://bmjopen.bmj.com/site/about/guidelines.xhtml

The $UPC^{GP\ list}$ index is significantly associated with acute hospital admissions and could be a possible quality measure to inform policy decisions and follow up policy changes if the goal is to ensure COC in primary care. COC at practice level could be estimated using national registry data as in the present paper or by data available within a practice, as Sidaway-Lee et al. describe (31). However, whether a maximal gain of COC is achieved, seems to depend on patient characteristics in addition to health care performance. This is an important finding in relation to current changes in several health care systems, which emphasize quick access, private service providers and private insurance-based services for groups that may afford to buy these services, at risk of losing benefit of COC and probably increased total health care costs. Altogether, it seems that the health care system needs a primary care organisation that facilitates COC but also patients who are informed about the benefit of COC and willing to use services so they might achieve COC; but there is need for further research on how to promote COC. Continuity of care seems to be especially important for patients with low education, and prioritisation of COC by GPs and health care policy might be a contribution to reducing inequalities in health. A main goal for the list patient system is to provide equitable health care for all inhabitants, and variation in continuity of care and the related consequences shown is unwarranted variation that should be reduced.

However, since our suggested index represents a new approach, it needs further assessment in research. It would be interesting to see if studies based on registry data from other contexts give similar results. Also, qualitative studies can give more in-depth understanding of various aspects of the COC concept and how patients and professionals view different COC measures. If survey information can be merged with registry data on health care utilization and GP lists, it should be possible to compare patients' reports with the two COC measures.

For peer review only - http://bmjopen.bmj.com/site/about/guidelines.xhtml

GPs' personal responsibilities are extensive and work pressure is high. The expectation to deliver continuous care with high accessibility is increasingly problematic when recruiting and maintaining GPs (32, 33). Our findings indicate that GPs organised in group practices might achieve the same benefits of COC for their patients as a longitudinal, personal continuity with one regular GP, at least regarding the number of acute hospital admissions. This finding might support a stronger focus on common responsibilities between GPs in the future, but changes should be followed by more research on effects.

Footnotes

- Contributors: THH and KM outlined the study in the application for the funding grant. Together with ØH they further developed the project idea. THH had the main responsibility for analysing data, and all authors contributed to the interpretation of data. ØH and KM drafted the article, all authors reviewed and edited the manuscript, and approved the version that is submitted. The corresponding author attests that all listed authors meet authorship criteria and that no others meeting the criteria have been omitted.
- Funding: This study is part of a project funded by The Research Council of Norway, project number 288592
- Competing interests: All authors have completed the ICMJE uniform disclosure form at www.icmje.org/coi_disclosure.pdf and declare to have no conflict of interests
- Ethical approval: The study is approved by Committee for Medical and Health Research Ethics (Project number 2019/771/REKvest). The ethics committee waived the need for patient consent for the use of these registry data. Data were linked by Statistics Norway and pseudo-anonymised before delivered to the researchers,

For peer review only - http://bmjopen.bmj.com/site/about/guidelines.xhtml

- Data sharing: Data are available from the national registries on a reasonable request. The use of data in this study is approved by application for this specific project only and data cannot be shared with anyone outside this project.
- The manuscript's guarantor (ØH) affirms that the manuscript is an honest, accurate, and transparent account of the study being reported; that no important aspects of the study have been omitted.
- Dissemination to participants and related patient and public communities: We are not able to disseminate the results of the research directly to study participants because the data used were anonymised, but plan to disseminate to community through general media general after the scientific publication.
- Patient and Public Involvement subsection: Patients or Public were not involved in the design or interpretation of this study
- Provenance and peer review: Not commissioned; externally peer reviewed.

For peer review only - http://bmjopen.bmj.com/site/about/guidelines.xhtml

References:

1. World Health O, United Nations Children's F. A vision for primary health care in the 21st century: towards universal health coverage and the Sustainable Development Goals. Geneva: World Health Organization; 2018.
2. Pollack CE, Hussey PS, Rudin RS, Fox DS, Lai J, Schneider EC. Measuring care continuity: a comparison of claims-based methods. Med Care. 2016;54(5):e30.
3. Saultz JW. Defining and Measuring Interpersonal Continuity of Care. Ann Fam Med. 2003;1(3):134-43.
4. Uijen AA, Schers HJ, van Weel C. Continuity of care preferably measured from the patients' perspective. J Clin Epidemiol. 2010;63(9):998-9.
5. Haggerty JL, Reid RJ, Freeman GK, Starfield BH, Adair CE, McKendry R. Continuity of care: a multidisciplinary review. BMJ. 2003;327(7425):1219-21.
6. Gray DJP, Sidaway-Lee K, White E, Thorne A, Evans PH. Continuity of care with doctors—a matter of life and death? A systematic review of continuity of care and mortality. BMJ open. 2018;8(6):e021161.
7. Baker R, Freeman GK, Haggerty JL, Bankart MJ, Nockels KH. Primary medical care continuity and patient mortality: a systematic review. Br J Gen Pract. 2020;70(698):e600-e11.
8. van Walraven C, Oake N, Jennings A, Forster AJ. The association between continuity of care and outcomes: a systematic and critical review. J Eval Clin Pract. 2010;16(5):947-56.
9. Nyweide DJ, Anthony DL, Bynum JPW, Strawderman RL, Weeks WB, Casalino LP, et al. Continuity of Care and the Risk of Preventable Hospitalization in Older Adults. JAMA Internal Medicine. 2013;173(20):1879-85.
10. Kohnke H, Zielinski A. Association between continuity of care in Swedish primary care and emergency services utilisation: a population-based cross-sectional study. Scand J Prim Health Care. 2017;35(2):113-9.
11. Freund T, Campbell SM, Geissler S, Kunz CU, Mahler C, Peters-Klimm F, et al. Strategies for reducing potentially avoidable hospitalizations for ambulatory care–sensitive conditions. Ann Fam Med. 2013;11(4):363-70.
12. Caminal J, Starfield B, Sánchez E, Casanova C, Morales M. The role of primary care in preventing ambulatory care sensitive conditions. Eur J Public Health. 2004;14(3):246-51.
13. Barker I, Steventon A, Deeny SR. Association between continuity of care in general practice and hospital admissions for ambulatory care sensitive conditions: cross sectional study of routinely collected, person level data. BMJ. 2017;356.
14. Katz DA, McCoy KD, Vaughan-Sarrazin MS. Does Greater Continuity of Veterans Administration Primary Care Reduce Emergency Department Visits and Hospitalization in Older Veterans? J Am Geriatr Soc. 2015;63(12):2510-8.
15. Bentler SE, Morgan RO, Virnig BA, Wolinsky FD. The association of longitudinal and interpersonal continuity of care with emergency department use, hospitalization, and mortality among Medicare beneficiaries. PLoS One. 2014;9(12):e115088-e.
16. Ministry of Health and Care Services. Regulation relating to a Municipal Regular GP Scheme. Oslo.2000 [Available from: http://www.regjeringen.no/en/dep/hod/dok/lover_regler/reglement/2000/regulation-relating-to-a-municipal-regul.html?id=420530.
17. Breslau N, Reeb KG. Continuity of care in a university-based practice. Acad Med. 1975;50(10):965-9.
18. Lustman A, Comaneshter D, Vinker S. Interpersonal continuity of care and type two diabetes. Prim Care Diabetes. 2016;10(3):165-70.

For peer review only - http://bmjopen.bmj.com/site/about/guidelines.xhtml

19. Boulton M, Tarrant C, Windridge K, Baker R, Freeman GK. How are different types of continuity achieved? A mixed methods longitudinal study. Br J Gen Pract. 2006;56(531):749-55.
20. Nutting PA, Goodwin MA, Flocke SA, Zyzanski SJ, Stange KC. Continuity of primary care: to whom does it matter and when? Ann Fam Med. 2003;1(3):149-55.
21. Norwegian Directorate of Health. Sammenstilling av data fra allmennlegetjenesten. Oslo.2019 [Available from: https://www.helsedirektoratet.no/rapporter/sammenstilling-av-data-fra-allmennlegetjenesten.
22. Forman JH, Robinson CH, Krein SL. Striving toward team-based continuity: provision of same-day access and continuity in academic primary care clinics. BMC Health Serv Res. 2019;19(1):145.
23. Bardsley M, Blunt I, Davies S, Dixon J. Is secondary preventive care improving? Observational study of 10-year trends in emergency admissions for conditions amenable to ambulatory care. BMJ Open. 2013;3(1).
24. Gunasekara FI, Richardson K, Carter K, Blakely T. Fixed effects analysis of repeated measures data. Int J Epidemiol. 2014;43(1):264-9.
25. Steventon A, Deeny S, Friebel R, Gardner T, Thorlby R. Briefing: emergency hospital admissions in England: which may be avoidable and how? The Health Foundation. 2018.
26. Pereira Gray DJ, Sidaway-Lee K, White E, Thorne A, Evans PH. Continuity of care with doctors—a matter of life and death? A systematic review of continuity of care and mortality. BMJ Open. 2018;8(6):e021161.
27. Honeyford K, Baker R, Bankart MJG, Jones D. Modelling factors in primary care quality improvement: a cross-sectional study of premature CHD mortality. BMJ Open 2013;3(10).
28. Levene LS, Bankart J, Khunti K, Baker R. Association of primary care characteristics with variations in mortality rates in England: an observational study. PLoS One. 2012;7(10):e47800.
29. Donaldson MS. Continuity of care: a reconceptualization. Med Care Res Rev. 2001;58(3):255-90.
30. Schers H, Webster S, van den Hoogen H, Avery A, Grol R, van den Bosch W. Continuity of care in general practice: a survey of patients' views. Br J Gen Pract. 2002;52(479):459-62.
31. Sidaway-Lee K, Gray DP, Evans P. A method for measuring continuity of care in day-to-day general practice: a quantitative analysis of appointment data. Br J Gen Pract. 2019;69(682):e356-e62.
32. Baird B, Charles A, Honeyman M, Maguire D, Das P. Understanding pressures in general practice. The King's Fund. 2016.
33. Ministry of Health and Care Services. Handlingsplan for allmennlegetjenesten. Attraktiv, kvalitetssikker og teambasert 2020-2024. Oslo.2020 [Available from: https://www.regjeringen.no/contentassets/96f6581333ee48559cdabf23c8772294/handlingsplan-for-allmennleger.pdf.

For peer review only - http://bmjopen.bmj.com/site/about/guidelines.xhtml

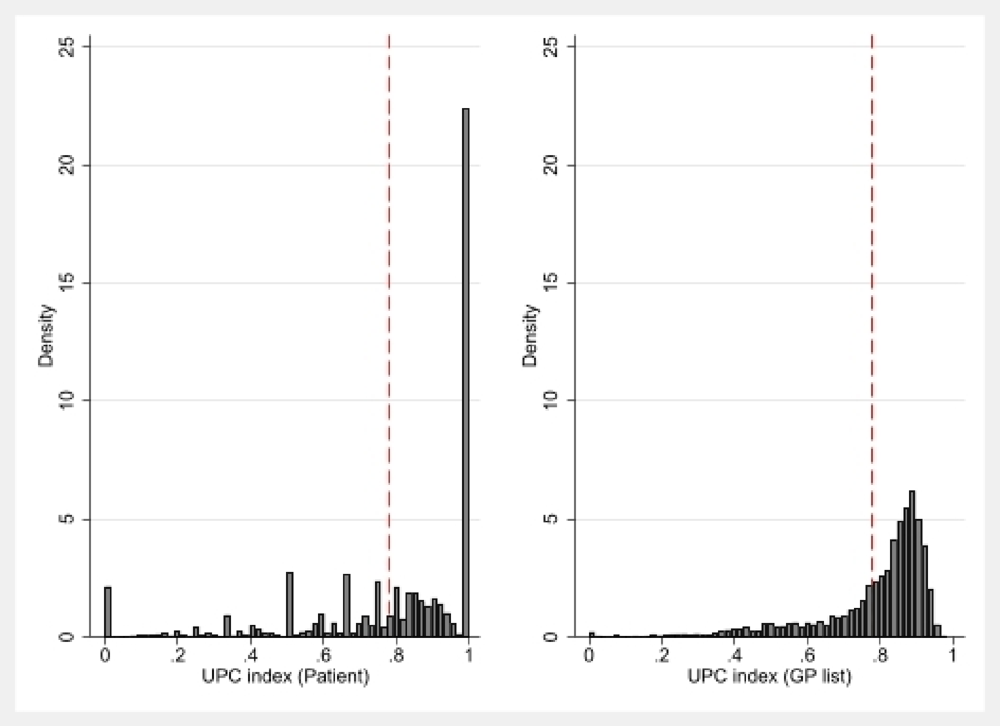

139x101mm (96 x 96 DPI)

Supplemenary file A

Supplementary file A shows the estimated association between the three outcomes and the UPC$^{GP\ list}$ continuity index, using a piecewise linear function for age, income, number of GP visits, and number of hospital admissions (linear splines). All models were estimated with three knots at the 25th, 50th and 75th percentile for the continues variables.

Appendix table 1. Linear splines

	(1) Emergency admission	(2) Emergency admission for ACSC	(3) Mortality
Index score	-0.025***	-0.006*	0.000
	(-3.43)	(-1.99)	(0.35)
N	757873	757873	757873
R^2	0.092	0.051	0.050

t statistics in parentheses. Standard errors clustered at GP level.
In all estimations, control variables include individual patient characteristics listed in table 1 and municipality-fixed effects.
* $p < 0.05$, ** $p < 0.01$, *** $p < 0.001$

For peer review only

The RECORD statement – checklist of items, extended from the STROBE statement, that should be reported in observational studies using routinely collected health data.

	Item No.	STROBE items	Location in manuscript where items are reported	RECORD items	Location in manuscript where items are reported
**Title and abstract**					
	1	(a) Indicate the study's design with a commonly used term in the title or the abstract (b) Provide in the abstract an informative and balanced summary of what was done and what was found		RECORD 1.1: The type of data used should be specified in the title or abstract. When possible, the name of the databases used should be included.<br><br>RECORD 1.2: If applicable, the geographic region and timeframe within which the study took place should be reported in the title or abstract.<br><br>RECORD 1.3: If linkage between databases was conducted for the study, this should be clearly stated in the title or abstract.	Abstract<br><br>Abstract<br><br>Abstract
**Introduction**					
Background rationale	2	Explain the scientific background and rationale for the investigation being reported			Background
Objectives	3	State specific objectives, including any prespecified hypotheses			Background page 5-6
**Methods**					
Study Design	4	Present key elements of study design early in the paper			Page 7-8
Setting	5	Describe the setting, locations, and relevant dates, including periods of recruitment, exposure, follow-up, and data collection			Page 7-8

For peer review only

Participants	6	*(a) Cohort study* - Give the eligibility criteria, and the sources and methods of selection of participants. Describe methods of follow-up<br>*Case-control study* - Give the eligibility criteria, and the sources and methods of case ascertainment and control selection. Give the rationale for the choice of cases and controls<br>*Cross-sectional study* - Give the eligibility criteria, and the sources and methods of selection of participants<br><br>*(b) Cohort study* - For matched studies, give matching criteria and number of exposed and unexposed<br>*Case-control study* - For matched studies, give matching criteria and the number of controls per case		RECORD 6.1: The methods of study population selection (such as codes or algorithms used to identify subjects) should be listed in detail. If this is not possible, an explanation should be provided.<br><br>RECORD 6.2: Any validation studies of the codes or algorithms used to select the population should be referenced. If validation was conducted for this study and not published elsewhere, detailed methods and results should be provided.<br><br>RECORD 6.3: If the study involved linkage of databases, consider use of a flow diagram or other graphical display to demonstrate the data linkage process, including the number of individuals with linked data at each stage.	Page 8-9 "sample selection"<br><br>Use complete population data, major selection is registered contacts, that is a valid variable in the national databases for health care use.<br><br>We have given a details description in page 8
Variables	7	Clearly define all outcomes, exposures, predictors, potential confounders, and effect modifiers. Give diagnostic criteria, if applicable.		RECORD 7.1: A complete list of codes and algorithms used to classify exposures, outcomes, confounders, and effect modifiers should be provided. If these cannot be reported, an explanation should be provided.	Described in sample section page 8 and following pages describing variables
Data sources/ measurement	8	For each variable of interest, give sources of data and details of methods of assessment (measurement).<br>Describe comparability of assessment methods if there is more than one group			Page 9-11

For peer review only - http://bmjopen.bmj.com/site/about/guidelines.xhtml

Bias	9	Describe any efforts to address potential sources of bias			
Study size	10	Explain how the study size was arrived at			Use complete population as source and database population
Quantitative variables	11	Explain how quantitative variables were handled in the analyses. If applicable, describe which groupings were chosen, and why			See "Variables" and "Statistical analyses" page 9-11
Statistical methods	12	(a) Describe all statistical methods, including those used to control for confounding<br>(b) Describe any methods used to examine subgroups and interactions<br>(c) Explain how missing data were addressed<br>(d) *Cohort study* - If applicable, explain how loss to follow-up was addressed<br>*Case-control study* - If applicable, explain how matching of cases and controls was addressed<br>*Cross-sectional study* - If applicable, describe analytical methods taking account of sampling strategy<br>(e) Describe any sensitivity analyses			Statistical methods are described page 10-11
Data access and cleaning methods		..		RECORD 12.1: Authors should describe the extent to which the investigators had access to the database population used to create the study population.	Use complete national register data as source, se page 7-8

For peer review only - http://bmjopen.bmj.com/site/about/guidelines.xhtml

				RECORD 12.2: Authors should provide information on the data cleaning methods used in the study.	Register use strict control on in-data. See also page7-8
Linkage		..		RECORD 12.3: State whether the study included person-level, institutional-level, or other data linkage across two or more databases. The methods of linkage and methods of linkage quality evaluation should be provided.	Linking described pages 7-8
**Results**					
Participants	13	(a) Report the numbers of individuals at each stage of the study (*e.g.*, numbers potentially eligible, examined for eligibility, confirmed eligible, included in the study, completing follow-up, and analysed)<br>(b) Give reasons for non-participation at each stage.<br>(c) Consider use of a flow diagram		RECORD 13.1: Describe in detail the selection of the persons included in the study (*i.e.,* study population selection) including filtering based on data quality, data availability and linkage. The selection of included persons can be described in the text and/or by means of the study flow diagram.	Page 11, selection procedures described in Methods
Descriptive data	14	(a) Give characteristics of study participants (*e.g.*, demographic, clinical, social) and information on exposures and potential confounders<br>(b) Indicate the number of participants with missing data for each variable of interest<br>(c) *Cohort study* - summarise follow-up time (*e.g.*, average and total amount)			Table 1
Outcome data	15	*Cohort study* - Report numbers of outcome events or summary measures over time			Table 2

For peer review only

		Case-control study - Report numbers in each exposure category, or summary measures of exposure<br>*Cross-sectional study* - Report numbers of outcome events or summary measures			
Main results	16	(a) Give unadjusted estimates and, if applicable, confounder-adjusted estimates and their precision (e.g., 95% confidence interval). Make clear which confounders were adjusted for and why they were included<br>(b) Report category boundaries when continuous variables were categorized<br>(c) If relevant, consider translating estimates of relative risk into absolute risk for a meaningful time period			Table 2 Unadjusted estimates are not given, to be able to present different models in the same tables.
Other analyses	17	Report other analyses done—e.g., analyses of subgroups and interactions, and sensitivity analyses			See "Statistical analyses" and table 3 and 4
**Discussion**					
Key results	18	Summarise key results with reference to study objectives			Page 18
Limitations	19	Discuss limitations of the study, taking into account sources of potential bias or imprecision. Discuss both direction and magnitude of any potential bias		RECORD 19.1: Discuss the implications of using data that were not created or collected to answer the specific research question(s). Include discussion of misclassification bias, unmeasured confounding, missing data, and changing eligibility over time, as they pertain to the study being reported.	Page 21

For peer review only

Interpretation	20	Give a cautious overall interpretation of results considering objectives, limitations, multiplicity of analyses, results from similar studies, and other relevant evidence			Page 22-23
Generalisability	21	Discuss the generalisability (external validity) of the study results			Page 21
**Other Information**					
Funding	22	Give the source of funding and the role of the funders for the present study and, if applicable, for the original study on which the present article is based			At the end of Methods
Accessibility of protocol, raw data, and programming code		..		RECORD 22.1: Authors should provide information on how to access any supplemental information such as the study protocol, raw data, or programming code.	The data are available from the national registers. Programming code be given by authors

*Reference: Benchimol EI, Smeeth L, Guttmann A, Harron K, Moher D, Petersen I, Sørensen HT, von Elm E, Langan SM, the RECORD Working Committee. The REporting of studies Conducted using Observational Routinely-collected health Data (RECORD) Statement. *PLoS Medicine* 2015; in press.

*Checklist is protected under Creative Commons Attribution (CC BY) license.

For peer review only

Continuity of care, measurement and association with hospital admission and mortality – a registry-based longitudinal cohort study

Journal:	*BMJ Open*
Manuscript ID	bmjopen-2021-051958.R2
Article Type:	Original research
Date Submitted by the Author:	18-Oct-2021
Complete List of Authors:	Hetlevik, Øystein; University of Bergen, Department of Global Public Health and Primary Care<br>Holmås, Tor ; NORCE Norwegian Research Centre AS Research Area Social Sciences<br>Monstad, Karin; NORCE Norwegian Research Centre AS Research Area Social Sciences
**Primary Subject Heading**:	General practice / Family practice
Secondary Subject Heading:	Health services research
Keywords:	Organisation of health services < HEALTH SERVICES ADMINISTRATION & MANAGEMENT, PRIMARY CARE, Quality in health care < HEALTH SERVICES ADMINISTRATION & MANAGEMENT

SCHOLARONE™
Manuscripts

I, the Submitting Author has the right to grant and does grant on behalf of all authors of the Work (as defined in the below author licence), an exclusive licence and/or a non-exclusive licence for contributions from authors who are: i) UK Crown employees; ii) where BMJ has agreed a CC-BY licence shall apply, and/or iii) in accordance with the terms applicable for US Federal Government officers or employees acting as part of their official duties; on a worldwide, perpetual, irrevocable, royalty-free basis to BMJ Publishing Group Ltd ("BMJ") its licensees and where the relevant Journal is co-owned by BMJ to the co-owners of the Journal, to publish the Work in this journal and any other BMJ products and to exploit all rights, as set out in our licence.

The Submitting Author accepts and understands that any supply made under these terms is made by BMJ to the Submitting Author unless you are acting as an employee on behalf of your employer or a postgraduate student of an affiliated institution which is paying any applicable article publishing charge ("APC") for Open Access articles. Where the Submitting Author wishes to make the Work available on an Open Access basis (and intends to pay the relevant APC), the terms of reuse of such Open Access shall be governed by a Creative Commons licence – details of these licences and which Creative Commons licence will apply to this Work are set out in our licence referred to above.

Other than as permitted in any relevant BMJ Author's Self Archiving Policies, I confirm this Work has not been accepted for publication elsewhere, is not being considered for publication elsewhere and does not duplicate material already published. I confirm all authors consent to publication of this Work and authorise the granting of this licence.

For peer review only

Continuity of care, measurement and association with hospital admission and mortality – a registry-based longitudinal cohort study

Øystein Hetlevik, Professor, Department of Global Public Health and Primary Care, University of Bergen, Bergen, Norway

Tor Helge Holmås, Research Professor, NORCE Norwegian Research Centre, Bergen, Norway

Karin Monstad, Research Professor, NORCE Norwegian Research Centre, Bergen, Norway

Corresponding author:
Øystein Hetlevik
E-mail: oystein.hetlevik@uib.no
Postal address:
Department of Global Public Health and Primary Care, University of Bergen,
Post-box 7804
N-5020 Bergen,
Norway

For peer review only

ABSTRACT

Objective To assess whether continuity of care (COC) with a general practitioner (GP) is associated with mortality and hospital admissions for older patients We argue that the conventional continuity measure may overestimate these associations. To better reflect COC as a GP quality indicator, we present an alternative, service-based measure.

Design Registry-based, population-level longitudinal cohort study.

Setting Linked data from Norwegian administrative health care registries, including 3,989 GPs.

Participants 757,873 patients aged 60–90 years with ≥2 contacts with a GP during 2016 and 2017.

Main outcome measure All-cause emergency hospital admissions, emergency admissions for ambulatory care sensitive conditions, and mortality, in 2018.

Results We assessed continuity of care using the conventional usual provider of care index ($UPC^{patient}$) and an alternative/supplementary index ($UPC^{GP\ list}$) based on the COC for all other patients enlisted with the same preferred GP.

For both indices, the mean index score was 0.78. Our model controls for demographic and socio-economic characteristics, prior health care use, and municipality-fixed effects. Overall, $UPC^{GP\ list}$ shows a much weaker association between COC and the outcomes. For both indices, there is a negative relationship between COC and hospital admissions. A 0.2-point increase in the index score would reduce ambulatory care sensitive conditions by 8.1% (CI 7.1–9.1) versus merely 1.9% (0.2–3.5) according to $UPC^{patient}$ and $UPC^{GP\ list}$, respectively. Using $UPC^{GP\ list}$, we find that mortality is no longer associated with COC. There was greater evidence for an association between COC and all-cause admissions among patients with low education.

Conclusions

A continuity measure based on each patient’s contacts with own preferred GP may overestimate the importance of COC as a feature of the GP practice. An alternative, service-based measure of continuity could be suitable as a quality measure in primary health care.

For peer review only - http://bmjopen.bmj.com/site/about/guidelines.xhtml

Facilitating continuity should be considered a health policy measure to reduce inequalities in health.

Strengths and limitations of this study

- This study is based on detailed nationwide registry data at the patient level for the entire population aged 60-90 years, including all GP consultations and hospital admissions.
- The analysis presents a novel service-based continuity of care index, measured at GP practice level, and discusses how this measure relates to the conventional usual provider of care index.
- We analyse how associations vary across gender and levels of education, and how different forms of breaches of COC are associated with patient outcomes.
- A limitation is lack of detailed information of each patient's severity of disease, although we partly compensate for this by including prior health care use as a proxy for patients' health status.
- Our data sources do not capture patient experiences with and evaluation of COC.

For peer review only

For peer review only - http://bmjopen.bmj.com/site/about/guidelines.xhtml

Introduction

Continuity of care (COC) is a declared goal in primary care systems (1). The concept of COC is broad, but most often refers to personal continuity, either measured longitudinally or by standardised questionnaires eliciting information on patient-provider relational continuity (2-4). Additionally, the continuity concept includes informational continuity and management continuity as measures of level of seamless services across different care providers or sites (5). In this study, COC reflects the extent to which GP contacts are concentrated to the same GP in a given time period, which serves as a proxy for relational continuity. Recent literature reviews conclude that continuity is associated with lower mortality (6, 7) and a reduction in hospital admissions (8), indicating better patient treatment in primary care and lower total health care costs (9, 10). For chronic conditions that are considered manageable in primary care (ambulatory care sensitive conditions, ACSC), hospitalisations are considered a particularly sensitive marker of primary care quality (11, 12). Barker et al. (13) showed that longitudinal COC was associated with reduced number of hospital admissions for ACSCs. Despite the acknowledged virtues of continuity of care, there is a steadily increasing policy drive towards giving priority to swift access to care (14), which may contradict the possibility of seeing a preferred physician and challenge the goal of continuity of care (13, 15).

Since 2001, more than 99% Norwegian inhabitants have been assigned a regular GP in a list patient system, and as such have a defined usual provider, while also having the option to switch GP twice in a calendar year. Improving continuity in the GP-patient relationship was one of the main arguments for this reform (16).

In this study, we contribute to the existing literature in several respects. First and foremost, we critically assess the conventional usual provider of care index, which we argue may overestimate the association between emergency hospital admissions and COC as a feature of

For peer review only - http://bmjopen.bmj.com/site/about/guidelines.xhtml

the GP service. The usual provider of care index represents, for each patient, the proportion of contacts with a preferred provider, most often a GP, in relation to all contacts during a given period (17). Our argument is that the conventional usual provider of care index disregards the fact that when the patient visits another GP than his/her preferred GP, he/she is in many cases in need of acute medical care, as discussed by Lustman et al. (18). Patients in acute need of health care are more inclined to be hospitalised, therefore patient unobserved health is a confounding factor that most likely leads to an overestimation of the (presumably negative) association between COC and hospital emergency admissions. Also, patients may prioritise differently between swift access to any GP and continuity of care with their regular GP, which may influence their relational COC independently of the COC offered at GP service level (19). We suggest an alternative measure, where COC is disentangled from each individual's unobserved health or personal choices. For each patient, we compute the continuity of care experienced by other patients having the same preferred GP. Our approach is detailed in the methods section.

Second, few studies have investigated how the importance of COC varies by patient characteristics (7), although COC seems to be of particular importance for patients in vulnerable groups (20). We measure the association between COC and outcomes for patients with different education levels. Third, we investigate how hospital admission and mortality are associated with different forms of discontinuity of care, where the patients instead of using the preferred GP either use another GP at ordinary opening hours or out-of-hours (OOH) services.

Since 95% of all Norwegian GPs work in group practices (21), patients may have relational continuity to several GPs who also share electronic health records. This facilitates informational and management continuity when the preferred GP is not available (22). In contrast, at OOH services, patients meet an unfamiliar physician with no access to the

For peer review only - http://bmjopen.bmj.com/site/about/guidelines.xhtml
For peer review only

preferred GP's patient records, with low possibility for informational or management continuity to cover up for the broken relational continuity. It is not clear how informational and management continuity is associated with hospital admissions (8). Our data allow us to investigate how different kinds of discontinuity of care is associated with hospital admissions as well as mortality. Finally, to our knowledge, this is the first analysis of COC that benefits from patient and GP level data for the entire population.

To sum up, our first aim is to assess whether COC with a GP is associated with mortality and hospital admissions for older patients. We focus on capturing how GP accessibility influences the association and present a GP service-based measure. Further, we aim to explore how the associations between the outcomes and the service-based COC measure vary across gender and educational level. Finally, we aim to analyse how these associations are influenced by breaches of personal COC compared with breaches of personal as well as informational COC.

Methods

Observational period

Several previous studies, e.g. Barker et al. (13), measure COC and the outcome within the same period. However, following Katz et al. (14), our empirical strategy is measuring continuity over a baseline period of two years (in our case, 2016–2017) before a follow-up period of one year (2018), when we measure the outcomes.

In this way, we avoid a potential reverse causality problem that arises because hospitalisation may lead to an increase in GP visits shortly after hospital discharge. If these visits are with own GP, COC for the relevant period will be measured as high, and the presumably negative association between COC and hospital admission will be washed out. Vice versa, if post-

For peer review only - http://bmjopen.bmj.com/site/about/guidelines.xhtml

discharge visits are with another GP, the negative relationship between COC and hospitalisation will be overestimated.

In addition, when applying a baseline period separated from a follow-up period, we acknowledge that greater COC can lead to better disease control and delay or prevent the onset of end-stage disease.

Data sources

The analysis benefits from administrative registry data at the patient level, covering the entire population, with information on all GP visits in Norway (the KUHR registry). Data originate from reimbursement claims sent by the primary care physician following each consultation, whether at the GP's office or OOH visits at a primary care emergency centre. Since the KUHR registry includes patient identifiers and the consultation date, we can observe, for each patient, the number of primary physician visits during a given period. Furthermore, since the KUHR data also contain GP identifiers, they can be merged with information on each GP's patient list from the Regular GP database. This allows us to identify whether the visit was with the patient's own GP, another GP or at an OOH service.

Data on hospital admissions are provided by the Norwegian Patient Registry (NPR) and include information on all hospitalisations in Norway: the date of the admission, whether the admission was planned or unplanned, inpatient or outpatient, and diagnoses according to the ICD-10 system.

From Statistics Norway we obtained data on patient characteristics, including age, sex, education, income, whether the individual lives in a one-person household, and date of death.

Sample selection

For peer review only - http://bmjopen.bmj.com/site/about/guidelines.xhtml

Since COC is regarded especially important for patients with chronic conditions, which are more widespread in the elderly population, we restrict the sample to the age group 60–90. The number of inhabitants in this age group by January 1, 2016 is 1,100,010, of which nearly all, 1,099,268, are registered with a regular GP, while the group of regular GPs at this point counts 4,644. We apply the following sample selection criteria:

- the sample is restricted to patients who belong to the same GP-patient list in the baseline period. This criterion ensures that a unique preferred GP can be defined (in total 947,153 patients) and implies that we only include GPs practising by January 1, 2016 and throughout the baseline period (3,990 GPs).
- we exclude patients who die within the baseline period (53,366 patients).
- we restrict the sample to patients who have at least two consultations with primary care physicians during the baseline period (in total 757,873 patients and 3,989 GPs). This is in line with most previous studies using UPC as a measure of continuity (2, 13).

Outcomes

Our dependent variables are three-fold, and all outcomes are measured in 2018: (i) the number of *all-cause acute hospital admissions,* (ii) the number of *acute hospital admissions for ACSCs*, where the definition of ACSC is adapted from Bardsley et al. (23), also applied in Barker et al. (13), (iii) *mortality*, a variable that equals 1 if the individual dies during 2018.

Continuity of care measures

We apply two alternative definitions of the provider of care index, labelled $UPC^{patient}$ and $UPC^{GP\ list}$. Notably, in accordance with Norwegian health care policy, we define "the usual provider" as the regular GP that the patient is enlisted with. The $UPC^{patient}$ index is the

For peer review only - http://bmjopen.bmj.com/site/about/guidelines.xhtml

traditional index applied in for instance Barker et al. (13). For instance, if an individual has 10 contacts in total, and 6 of them are with his own GP, the $UPC^{patient}$ score will be 0.6.

In order to disentangle the COC measure from individual *i'* s unobserved health and background characteristics (as explained in the introduction), we construct continuity measure $UPC^{GP\ list}$. This index is based on the continuity of care experienced by *patients other than i* belonging to the same GP list population. That implies that for each patient, we use information on the continuity experienced by other patients enlisted with *i*'s GP. An example could be a group of three patients A, B, C enlisted with the same GP. If A has 8 out of 10 visits with own GP, B has 3 out of 7, and C has 12 out of 12, then A's $UPC^{GP\ list}$ index score would be (3+12)/(7+12), B's score would be (8+12)/(10+12) and C's score would be (8+3)/(10+7). The UPC indices are in the interval (0,1), with a higher score reflecting greater continuity of care.

Formally, the $UPC^{GP\ list}$ index can be expressed as follows:

$$UPC_i^{GP\ list} = \frac{X_{j*}^{-i}}{\sum_{j=1}^{J} X_j^{-i}}$$

where *i* is an individual, *j* is one of *J* GPs, *j** is the GP whom individual *i* is enlisted with, and X_j^{-i} is the number of visits to GP *j* of all individuals enlisted with j*, except for *i*. Using this notation, the $UPC^{patient}$ index is defined as

$$UPC_i^{patient} = \frac{X_{j*}^{i}}{\sum_{j=1}^{J} X_j^{i}}$$

For peer review only - http://bmjopen.bmj.com/site/about/guidelines.xhtml

The $UPC^{GP\ list}$ index is a general concept, applicable irrespective of the size of the GP list population. If GP list populations are large, the $UPC^{GP\ list}$ index will be very similar to an index at GP level where all patients are included. We have estimated using such a simpler index, and results are virtually identical (not reported here).

When data availability allows it, the continuity of care analysis can include an investigation of the impact of different breaches of personal COC. For this purpose, we construct two indices of *dis*continuity: the proportion of all consultations in a GP list population that is with another GP at ordinary opening hours (Other-GP index) and the proportion of OOH visits (OOH - index), respectively.

Covariates

To control for patient heterogeneity, we include information on a number of individual demographic and socio-economic characteristics: sex, age, level of education in three categories (primary, secondary and higher education), gross total income in NOK 100 000, and an indicator for one-person household (measured in 2016). Furthermore, utilisation of health care in the baseline period (2016–2017) may be indicative of the individual's health status. As controls, we therefore include the number of GP visits, the number of all-cause hospital admissions (whether planned or unplanned), the proportion of inpatient admissions, as well as the proportion unplanned hospital admissions.

Statistical analysis

We used multivariable regression with municipality-fixed effects (the municipality where the GP practice is located), to test the association between COC and the outcomes, number of hospital admissions and mortality. To reduce bias due to population heterogeneity, we included a series of covariates that may influence the association: individual demographic and

For peer review only - http://bmjopen.bmj.com/site/about/guidelines.xhtml

socio-economic characteristics as well as health status indicators, as detailed above. Furthermore, a fixed effects approach can reduce the impact of unmeasured confounding. Since the fixed effects estimator relies only on variation within the chosen unit, this estimator is not affected by confounding from unmeasured time-invariant factors. Notice that the term "fixed effects" is distinct from that of the statistical literature, where this term typically is used in the context of random effects or mixed models describing a parameter associated with an entire population (see e.g Gunasekara et al. for a non-technical presentation of the fixed effects estimator and a comparison with mixed models) (24). In the context of this analysis, the (municipality-) fixed effect estimator controls for any time-invariant differences between municipalities, such as centrality, which may influence the availability of GPs – in particular, experienced GPs – as well as distance to hospital and other specialist health services. Since our outcomes are either count data (number of admissions) or binary (mortality), non-linear models such as count data models or logit models may seem appropriate. However, estimating a fixed-effects logit model resulted in numerical overflow (due to too many effective observations). Therefore, we have chosen to estimate linear fixed effect models rather than non-linear models without fixed effect.

In stratified analyses, we estimate the associations separately by gender and by level of education. In an additional estimation, we use the two indices of *dis*continuity as main explanatory variables, while otherwise, the model specification is kept unchanged.

The ordinal covariates, such as age and number of GP visits, are included in their natural units, apart from income, which enters in NOK 100 000s. As a sensitivity analysis, we have estimated linear splines models, which gave virtually identical results (for details, see Supplementary file A). The COC index enters the model linearly, which is common in the literature.

For peer review only - http://bmjopen.bmj.com/site/about/guidelines.xhtml

The relationship between COC and our outcomes is complex, and we do not claim that the current analysis detects causal pathways. For instance, it is conceivable that GPs offering greater COC attract a higher proportion of patients with chronic conditions, which would imply a selection bias. Still, we have tried to address the identification issues in several ways. First, we present an alternative index that we argue modifies the impact of patient unobserved health on observed COC. Second, information on a rich set of covariates helps reflect the individual's health status. Third, we observe these control variables in a baseline period *pre* outcomes. Finally, to improve inference, we take into account that patients enlisted with the same GP most likely share some (unobservable) characteristics that may influence outcomes, such as health, social background, and a preference for a certain GP practice style. These characteristics may not be fully adjusted for by municipality fixed effects, therefore, we cluster standard errors at the GP level.

Analyses were performed using STATA version 16.

Patient and public involvement

Patients or public were not involved in the design or interpretation of this study

Results

In total, 757,873 individuals, enlisted with 3,989 GPs, met our inclusion and exclusion criteria. In the following, we will compare results from using the conventional usual provider of care index to results from using the service-based continuity of care measure presented above.

Figure 1 below reveals that for these two indices, mean scores are similar, yet there are large differences in the distribution. Note that for 22.5% of the sample, the $UPC^{patient}$ score equals

For peer review only - http://bmjopen.bmj.com/site/about/guidelines.xhtml

one, i.e., for these patients, all their primary physician visits during the baseline period were with their own GP. Also, about 2.5 per cent of the sample had no visits to own GP, i.e., a UPC^{patient} score of zero. By construction, the values zero and one do not appear when we apply the UPC$^{GP\ list}$ index.

*** INSERT FIGURE 1 HERE*****

Figure 1. Distribution of usual provider of care indices (*n*=757,873) based on data from individual patients (Left figure: UPC^{patient}) and at GP practice level (right figure: UPC$^{GP\ list}$). Red dotted lines: mean value of each index.

Table 1 reports summary statistics of outcome variables and covariates. We see that 53 per cent of the sample are women, the mean age is 70.7 years and 35 per cent live alone. The overall use of health care is high, about 9.4 GP visits and 5.1 hospital admissions during the two years' baseline period. This is to be expected, given the high age and the fact that a certain (low) level of GP use is an inclusion criterion. In the baseline period, the mean number of emergency hospital admissions per year is about 0.3 (11.9 per cent of 5.1 hospital admissions were emergency cases during these two years), while the mean number of all-cause hospital admissions in the follow-up year is slightly higher, 0.357. For the two COC measures, we see that the mean values are very similar (0.78), but the standard deviation is larger for the UPC^{patient} index, as can also be seen from Figure 1.

Table 1. Descriptive statistics, patients aged 60–90 with at least two GP visits in 2016–2017. Proportions or means with standard errors in parentheses.

For peer review only - http://bmjopen.bmj.com/site/about/guidelines.xhtml

Continuity of care (measured at baseline, 2016–17):	
UPC$^{patient\ 2}$	0.788 (0.263)
UPC$^{GP\ list\ 2}$	0.780 (0.169)
Patient characteristics (measured at baseline, 2016–17):	
Proportion females	0.531
Age	70.675 (7.562)
Proportion low education[3]	0.259
Proportion medium education[3]	0.498
Proportion high education[3]	0.243
Total income[1] (100,000 NOK)	4.310 (5.067)
Proportion one-person household[3]	0.353
No. of GP visits	9.432 (7.967)
No. of hospital admissions	5.103 (9.884)
Proportion inpatient stays	0.093
Proportion emergency admissions	0.119
Number of patients	757,873

[1] ACSC: Ambulatory care sensitive conditions[2] UPC^{patient} and UPC$^{GP\ list}$: Usual provider care index measured for each patient and for each GP practice, respectively.[3] Measured in 2016.

Table 2. Associations between continuity of care and the outcomes emergency admissions to hospital and mortality. Estimated with two different indices, standard errors clustered at GP level.

	Emergency admission		Emergency admission for ACSC[1]		Mortality	
	UPC^{patient} index[2]	UPC $^{GP\ list}$ index[2]	UPC ^{patient} index[2]	UPC $^{GP\ list}$ index[2]	UPC ^{patient} index[2]	UPC $^{GP\ list}$ index[2]
Index score	-0.104***	-0.028**	-0.027***	-0.006*	-0.010***	0.000
	(-22.16)	(-3.78)	(-15.67)	(-2.51)	(-13.42)	(0.28)
R^2 (within)	0.088	0.088	0.049	0.049	0.046	0.043
Mean Y	0.357	0.357	0.068	0.068	0.024	0.024
Relative effect[3]	-5.8%	-1.6%	-8.1%	-1.9%	-8.3%	0.2%
N	757,873	757,873	757,873	757,873	757,873	757,873

t statistics in parentheses. * $p < 0.05$ ** $p < 0.01$, *** $p < 0.001$.
In all estimations, control variables include individual patient characteristics listed in table 1 and municipality-fixed effects. [1]ACSC: Ambulatory care sensitive conditions. [2]UPC^{patient} and UPC$^{GP\ list}$: Usual provider care index measured for each patient and for each GP practice, respectively. [3]Percentage change in outcome when the index increases by 0.2.

Irrespective of COC measure (Table 2), we find a negative relationship between COC and emergency admissions, and notably, the estimated association is far stronger with the UPC^{patient} than with the UPC$^{GP\ list}$. Using the latter index, no relationship between continuity and mortality emerges. The following illustrates the difference in magnitude of estimates: when the index score increases by 0.2, the number of emergency admissions for ACSC is reduced by 8.1 per cent when measured with the UPC^{patient} and by merely 1.9 per cent when

For peer review only - http://bmjopen.bmj.com/site/about/guidelines.xhtml

measured with UPC$^{GP\ list}$. As expected, the relationship between COC and emergency admissions is stronger for ACSC than for other conditions, irrespective of index used.

The coefficient estimates of the control variables show the same pattern across outcomes: the number of emergency admissions as well as mortality is higher for males than for females, increasing in age, decreasing in education, virtually independent of income, and higher for individuals living in one-person households than for married/cohabitating. Individuals who are frequent users of GP or hospital visits in the baseline period have a higher number of hospital admissions and a higher mortality risk the following year.

Heterogeneity

In the following, we will investigate whether the relationship between COC (measured by the UPC$^{GP\ list}$ index) and our outcomes depends on patient characteristics. When we estimate separately by gender, we get qualitatively the same results as reported in Table 2, although with lower levels of significance. There is no clear difference in results between men and women (we have included an interaction term between the COC measure and gender, and its coefficient is not statistically significant, not reported here). Next, we estimate separately by the three levels of education. Regarding mortality, we find no statistically significant association for any of the subsamples defined by educational level. We therefore focus on the relationship between COC and hospital admission.

Table 3. Associations between continuity of care and emergency admissions to hospital, stratified analyses by educational level. Estimated with the UPC$^{GP\ list}$ index and standard errors clustered at GP level.

For peer review only - http://bmjopen.bmj.com/site/about/guidelines.xhtml

	Emergency admission			Emergency admission for ACSC[1]		
	Primary education	Secondary education	Higher education	Primary education	Secondary education	Higher education
Index score	-0.065***	-0.028**	0.010	-0.009	-0.007*	-0.000
	(-4.42)	(-2.73)	(0.78)	(-1.43)	(-2.09)	(-0.18)
R^2	0.093	0.086	0.075	0.011	0.046	0.038
Mean Y	0.440	0.342	0.298	0.099	0.063	0.043
Relative effect[2]	-2.6%	-1.3%	0.5%	-1.6%	-1.9%	-0.3%
N	196,482	377,635	183,756	196,482	377,635	183,756

t statistics in parentheses. * $p < 0.05$, ** $p < 0.01$, *** $p < 0.001$.
Control variables include individual patient characteristics listed in table 1and municipality-fixed effects.
[1]ACSC: Ambulatory care sensitive conditions. [2]Percentage change in outcome of a one standard deviation change in the index score.

From Table 3 we learn that the association between COC and all-cause emergency admissions is far stronger for patients with primary education than for the two other categories. There is no such socio-economic gradient for the outcome emergency admissions for ACSC.

Inspecting different forms of discontinuity of care

So far, our explanatory variable of interest has been the continuity with which patients in a GP list population visit their own GP, while we have not distinguished between visits to other providers of primary doctor services. We now change the focus of the analysis, to investigate the relationship between the outcomes studied and different forms of *dis*continuity of care. In our data, we can identify two categories of such consultations: visits to another GP at ordinary opening hours (a partner, registrar or locum), and OOH visits. As reported in Table 1, about 79% of all consultations are with own GP, while about 17% are with another GP, and 5% are OOH visits.

In the following, we will compare outcomes for individuals belonging to list populations that, to different degrees, visit other GPs or OOH services, respectively. We find this interesting to investigate, since different forms of broken COC bear different implications for informational and management continuity and probably also relational continuity. In this part of the

For peer review only - http://bmjopen.bmj.com/site/about/guidelines.xhtml

analysis, we therefore change our specification by replacing our COC measure with two new explanatory variables: (i) Other-GP index (the proportion of GP visits that is with another GP than the preferred GP during ordinary opening hours), and (ii) the OOH-index (the proportion of GP visits that is OOH visits). Both proportions are out of the total number of GP and OOH doctor visits in the GP list population.

As before, we estimate outcomes at the individual level, controlling for municipality-fixed effects and individual patient characteristics related to the baseline period. Results are reported in Table 4.

Table 4. Associations between proportion of consultations with other GPs or OOH services in list population of the patient's regular GP and the emergency admissions to hospital and mortality.

	Emergency admission	Emergency admission for ACSC[1]	Mortality
Other-GP index	0.014^{*}	0.002	-0.000
	(1.99)	(0.71)	(-0.47)
OOH-index	0.959^{***}	0.285^{***}	0.017
	(9.57)	(7.45)	(1.49)
R^2	0.088	0.048	0.046
Mean Y	0.357	0.068	0.024
Relative effect variable "Other GP index"[2]	0.6%	0.5%	-0.2%
Relative effect variable «OOH-index» [2]	5.3%	8.3%	1.4%
N	757,873	757,873	757,873

t statistics in parentheses. * $p < 0.05$, ** $p < 0.01$, *** $p < 0.001$. Control variables include individual patient characteristics listed in table 1 and municipality-fixed effects. Standard errors clustered at GP level
[1]ACSC: Ambulatory care sensitive conditions. [2]Percentage change in outcome of a one standard deviation change in the index score.

We find that – for an individual patient in the list population – belonging to a list where OOH visits are more frequent is associated with a higher risk of both kinds of emergency admissions. On the other hand, emergency admissions for ASCS and mortality are both unrelated to the frequency (proportion) of consultations done by other GPs within their ordinary practice. We also find that GPs rarely treats patients belonging to a list outside their

For peer review only - http://bmjopen.bmj.com/site/about/guidelines.xhtml

group practice since such cases qualify for a particular fee, triggered in merely 0.6% of consultations.

Discussion

Main findings

In this nationwide registry-based study using individual-level data from Norwegian health care, we investigated COC as predictor of acute hospital admissions and mortality among patients aged 60–90 years. We compared two measures of COC: the conventional usual provider of care index ($UPC^{patient}$) and an index defined for each GP list population ($UPC^{GP\ list}$). In line with earlier studies, we find a marked association between $UPC^{patient}$ and lower rates for acute admissions and mortality. Both indices were inversely associated with all-cause emergency hospital admissions and emergency admissions for ACSC. However, the associations between COC and these outcomes were considerably weaker when COC was measured with the index defined at GP level. We found no association between mortality and the $UPC^{GP\ list}$ index, while there was an estimated 8.3% decrease in mortality with a reduction of 0.2 in the conventional usual provider of care index.

Analysing subsamples defined by level of education, we found a clear gradient with the strongest association between COC and all-cause hospital admissions for patients with low education. No such gradient was found for ACSC admissions.

Other things equal, emergency admissions are more common for individuals who belong to a GP list population where the use of OOH visits is frequent. We found no or minimal increase in number of emergency admissions if patients, instead of using their preferred GP, visited another GP. Data tell us that the “other GP” very rarely treats patients from outside the patient lists of his/her group practice. Therefore, we infer that, as a rule, “the other GP” and the preferred GP work within the same GP practice.

For peer review only - http://bmjopen.bmj.com/site/about/guidelines.xhtml

Interpretation of results

There is a policy drive towards reducing acute hospital admissions to reduce health care costs and streamline health services, and a well-functioning primary health care is judged to be important to achieve this (25). Earlier studies have found that COC is associated with a reduction in all-cause acute hospital admissions (10, 14) as well as acute admissions for ACSCs (13), and our results support these findings. Recent studies report an inverse association between COC and mortality (7, 26). In our study, we found this result only when using the conventional usual provider of care index. The lack of (a negative) association between COC at practice level and mortality partly contradicts earlier findings (27, 28).

The literature assessing the impact of COC on secondary health care utilisation mainly applies data on the individual patient and his/her use of the preferred GP. Our study supports the positive association between such measures of COC and positive health outcomes. However, in this paper, we add another perspective, by introducing an alternative index which reflects the COC in the preferred GP's practice population. Using this $UPC^{GP\ list}$ index, we found that the strength of COC as a predictor for admission attenuated markedly, and the $UPC^{GP\ list}$ index was not associated with mortality. These differences in associations between COC and the outcomes indicate that some patient characteristics or choices are reflected in the conventional usual provider of care index that add to the effect of COC as a measure of health care performance. Even though we adjust for prior care contacts in our analyses, variation in patient unobserved health may influence the $UPC^{patient}$ index. A patient with more unstable disease is more often in need of swift access to care and might therefore more often visit other GPs or OOH services, and more often need a hospital admission, as also discussed by Lustman et al. (18). The result is a lower conventional usual provider of care index and a higher frequency of admissions, thereby increasing the COC effects, partly independent of the

For peer review only - http://bmjopen.bmj.com/site/about/guidelines.xhtml

COC offered by the preferred GP during ordinary opening times. In addition, patients differ in their ability to manoeuvre in the health care system. Resourceful patients, who may also deal better with their diseases, may achieve a high COC even if the system level COC is low (19), and thereby also achieve the benefit of COC. This possibility is of course related to how the service is organised but will to a large extent reflect the patient's personal choices. A usual provider of care index based on the individual patient's contacts with health care might overestimate the effect of COC as a feature of the GP practice, and a service-based measure of COC might be a more useful marker of care quality.

Our finding that the strength of the association between COC and all-cause acute hospital admissions is inversely related to educational level possibly reflects socio-economic differences in ways of using health care. An explanation could be that patients with lower education tend to be more dependent on the GP facilitating COC to help them achieve the benefit of better continuity (20), in line with the theory of advocacy set forward by Donaldson (29). Also, multi-morbidity is more common among patients with low education, and based on previous studies, COC is of greater importance under such conditions (30). However, we found no educational gradient with respect to acute ACSC hospital admissions, which could indicate a more equally distributed GP service related to ACSC, with standard guidelines and scheduled follow-up programs.

The present study shows that the number of emergency hospital admissions is almost unaffected by other GPs taking a larger share of consultations in the list population. On the other hand, there was a marked increase in frequency of hospital admissions if the $UPC^{GP\ list}$ was lower due to more frequent use of OOH services by patients in the list population. This indicates that the benefits of COC can be reaped also when using other GPs in a group

For peer review only - http://bmjopen.bmj.com/site/about/guidelines.xhtml

practice, either because patients are familiar with more GPs ensuring relational continuity, or the lack of personal continuity can be compensated for by informational continuity (22). This finding supports a policy for increasing accessibility to GPs in daytime practices where a smaller group of GPs share responsibilities.

Strength and limitations

This analysis of COC is based on patient level data for the entire population, including all GP consultations and hospital admissions, combined with comprehensive data on patient characteristics and prior use of health care. In this analysis using claims data, health-related non-response is not a valid concern. Our findings are generalisable internationally in contexts where primary care is the first point of contact.

The longitudinal approach is also a strength, with COC estimation based on a two-year period prior to the one-year period when outcomes are assessed. A limitation is lack of information of each patient's severity of disease, although we partly compensate for this by including prior health care use as a proxy for health problems. A major weakness with registry data is that patient experiences with and evaluation of COC are not captured, whether at individual level or at GP list population level. In the discussion about the connection between COC and hospital admissions, an underlying assumption is that reduced admission rates is a positive outcome, especially for ACSC where there is a special potential to stabilise patients with good routine care, and hospital admission rates are considered a performance indicator of primary health care. Still, based on data in the present study, we cannot know if the reduced frequency of hospital admissions benefits the individual patient, except we observe no increase in mortality.

For peer review only - http://bmjopen.bmj.com/site/about/guidelines.xhtml

In this analysis, we compare the conventional COC index to an alternative index. There are several indices in use, which are highly correlated (2, 13). We chose the conventional usual provider of care index as a starting point precisely because it is often used, the most straightforward to interpret and possible to adapt to estimation of COC delivered in a health care organisation, in this case a GP practice. A COC measure that is more directly measuring performance of care, such as the proposed UPC^{GPlist} index, has a potential to be a useful quality measure, we argue.

Implications and further research

The $UPC^{GP\ list}$ index is significantly associated with acute hospital admissions and could be a possible quality measure to inform policy decisions and follow up policy changes if the goal is to ensure COC in primary care. COC at practice level could be estimated using national registry data as in the present paper or by data available within a practice, as Sidaway-Lee et al. describe (31). However, whether a maximal gain of COC is achieved, seems to depend on patient characteristics in addition to health care performance. This is an important finding in relation to current changes in several health care systems, which emphasize quick access, private service providers and private insurance-based services for groups that may afford to buy these services, at risk of losing benefit of COC and probably increased total health care costs. Altogether, it seems that the health care system needs a primary care organisation that facilitates COC but also patients who are informed about the benefit of COC and willing to use services so they might achieve COC; but there is need for further research on how to promote COC. Continuity of care seems to be especially important for patients with low education, and prioritisation of COC by GPs and health care policy might be a contribution to reducing inequalities in health. A main goal for the list patient system is to provide equitable

For peer review only - http://bmjopen.bmj.com/site/about/guidelines.xhtml

health care for all inhabitants, and variation in continuity of care and the related consequences shown is unwarranted variation that should be reduced.

However, since our suggested index represents a new approach, it needs further assessment in research. It would be interesting to see if studies based on registry data from other contexts give similar results. Also, qualitative studies can give more in-depth understanding of various aspects of the COC concept and how patients and professionals view different COC measures. If survey information can be merged with registry data on health care utilization and GP lists, it should be possible to compare patients' reports with the two COC measures.

GPs' personal responsibilities are extensive and work pressure is high. The expectation to deliver continuous care with high accessibility is increasingly problematic when recruiting and maintaining GPs (32, 33). Our findings indicate that GPs organised in group practices might achieve the same benefits of COC for their patients as a longitudinal, personal continuity with one regular GP, at least regarding the number of acute hospital admissions. This finding might support a stronger focus on common responsibilities between GPs in the future, but changes should be followed by more research on effects.

Footnotes

- Contributors: THH and KM outlined the study in the application for the funding grant. Together with ØH they further developed the project idea. THH had the main responsibility for analysing data, and all authors contributed to the interpretation of data. ØH and KM drafted the article, all authors reviewed and edited the manuscript, and approved the version that is submitted. The corresponding author attests that all listed authors meet authorship criteria and that no others meeting the criteria have been omitted.

For peer review only - http://bmjopen.bmj.com/site/about/guidelines.xhtml

- Funding: This study is part of a project funded by The Research Council of Norway, project number 288592
- Competing interests: All authors have completed the ICMJE uniform disclosure form at www.icmje.org/coi_disclosure.pdf and declare to have no conflict of interests
- Ethical approval: The study is approved by Committee for Medical and Health Research Ethics (Project number 2019/771/REKvest). The ethics committee waived the need for patient consent for the use of these registry data. Data were linked by Statistics Norway and pseudo-anonymised before delivered to the researchers,
- Data sharing: Data are available from the national registries on a reasonable request. The use of data in this study is approved by application for this specific project only and data cannot be shared with anyone outside this project.
- The manuscript's guarantor (ØH) affirms that the manuscript is an honest, accurate, and transparent account of the study being reported; that no important aspects of the study have been omitted.
- Dissemination to participants and related patient and public communities: We are not able to disseminate the results of the research directly to study participants because the data used were anonymised, but plan to disseminate to community through general media general after the scientific publication.
- Patient and Public Involvement subsection: Patients or Public were not involved in the design or interpretation of this study
- Provenance and peer review: Not commissioned; externally peer reviewed.

For peer review only - http://bmjopen.bmj.com/site/about/guidelines.xhtml

References:

1. World Health O, United Nations Children's F. A vision for primary health care in the 21st century: towards universal health coverage and the Sustainable Development Goals. Geneva: World Health Organization; 2018.
2. Pollack CE, Hussey PS, Rudin RS, Fox DS, Lai J, Schneider EC. Measuring care continuity: a comparison of claims-based methods. Med Care. 2016;54(5):e30.
3. Saultz JW. Defining and Measuring Interpersonal Continuity of Care. Ann Fam Med. 2003;1(3):134-43.
4. Uijen AA, Schers HJ, van Weel C. Continuity of care preferably measured from the patients' perspective. J Clin Epidemiol. 2010;63(9):998-9.
5. Haggerty JL, Reid RJ, Freeman GK, Starfield BH, Adair CE, McKendry R. Continuity of care: a multidisciplinary review. BMJ. 2003;327(7425):1219-21.
6. Gray DJP, Sidaway-Lee K, White E, Thorne A, Evans PH. Continuity of care with doctors—a matter of life and death? A systematic review of continuity of care and mortality. BMJ open. 2018;8(6):e021161.
7. Baker R, Freeman GK, Haggerty JL, Bankart MJ, Nockels KH. Primary medical care continuity and patient mortality: a systematic review. Br J Gen Pract. 2020;70(698):e600-e11.
8. van Walraven C, Oake N, Jennings A, Forster AJ. The association between continuity of care and outcomes: a systematic and critical review. J Eval Clin Pract. 2010;16(5):947-56.
9. Nyweide DJ, Anthony DL, Bynum JPW, Strawderman RL, Weeks WB, Casalino LP, et al. Continuity of Care and the Risk of Preventable Hospitalization in Older Adults. JAMA Internal Medicine. 2013;173(20):1879-85.
10. Kohnke H, Zielinski A. Association between continuity of care in Swedish primary care and emergency services utilisation: a population-based cross-sectional study. Scand J Prim Health Care. 2017;35(2):113-9.
11. Freund T, Campbell SM, Geissler S, Kunz CU, Mahler C, Peters-Klimm F, et al. Strategies for reducing potentially avoidable hospitalizations for ambulatory care–sensitive conditions. Ann Fam Med. 2013;11(4):363-70.
12. Caminal J, Starfield B, Sánchez E, Casanova C, Morales M. The role of primary care in preventing ambulatory care sensitive conditions. Eur J Public Health. 2004;14(3):246-51.
13. Barker I, Steventon A, Deeny SR. Association between continuity of care in general practice and hospital admissions for ambulatory care sensitive conditions: cross sectional study of routinely collected, person level data. BMJ. 2017;356.
14. Katz DA, McCoy KD, Vaughan-Sarrazin MS. Does Greater Continuity of Veterans Administration Primary Care Reduce Emergency Department Visits and Hospitalization in Older Veterans? J Am Geriatr Soc. 2015;63(12):2510-8.
15. Bentler SE, Morgan RO, Virnig BA, Wolinsky FD. The association of longitudinal and interpersonal continuity of care with emergency department use, hospitalization, and mortality among Medicare beneficiaries. PLoS One. 2014;9(12):e115088-e.
16. Ministry of Health and Care Services. Regulation relating to a Municipal Regular GP Scheme. Oslo.2000 [Available from: http://www.regjeringen.no/en/dep/hod/dok/lover_regler/reglement/2000/regulation-relating-to-a-municipal-regul.html?id=420530.
17. Breslau N, Reeb KG. Continuity of care in a university-based practice. Acad Med. 1975;50(10):965-9.
18. Lustman A, Comaneshter D, Vinker S. Interpersonal continuity of care and type two diabetes. Prim Care Diabetes. 2016;10(3):165-70.

For peer review only - http://bmjopen.bmj.com/site/about/guidelines.xhtml

19. Boulton M, Tarrant C, Windridge K, Baker R, Freeman GK. How are different types of continuity achieved? A mixed methods longitudinal study. Br J Gen Pract. 2006;56(531):749-55.
20. Nutting PA, Goodwin MA, Flocke SA, Zyzanski SJ, Stange KC. Continuity of primary care: to whom does it matter and when? Ann Fam Med. 2003;1(3):149-55.
21. Norwegian Directorate of Health. Sammenstilling av data fra allmennlegetjenesten. Oslo.2019 [Available from: https://www.helsedirektoratet.no/rapporter/sammenstilling-av-data-fra-allmennlegetjenesten.
22. Forman JH, Robinson CH, Krein SL. Striving toward team-based continuity: provision of same-day access and continuity in academic primary care clinics. BMC Health Serv Res. 2019;19(1):145.
23. Bardsley M, Blunt I, Davies S, Dixon J. Is secondary preventive care improving? Observational study of 10-year trends in emergency admissions for conditions amenable to ambulatory care. BMJ Open. 2013;3(1).
24. Gunasekara FI, Richardson K, Carter K, Blakely T. Fixed effects analysis of repeated measures data. Int J Epidemiol. 2014;43(1):264-9.
25. Steventon A, Deeny S, Friebel R, Gardner T, Thorlby R. Briefing: emergency hospital admissions in England: which may be avoidable and how? The Health Foundation. 2018.
26. Pereira Gray DJ, Sidaway-Lee K, White E, Thorne A, Evans PH. Continuity of care with doctors—a matter of life and death? A systematic review of continuity of care and mortality. BMJ Open. 2018;8(6):e021161.
27. Honeyford K, Baker R, Bankart MJG, Jones D. Modelling factors in primary care quality improvement: a cross-sectional study of premature CHD mortality. BMJ Open 2013;3(10).
28. Levene LS, Bankart J, Khunti K, Baker R. Association of primary care characteristics with variations in mortality rates in England: an observational study. PLoS One. 2012;7(10):e47800.
29. Donaldson MS. Continuity of care: a reconceptualization. Med Care Res Rev. 2001;58(3):255-90.
30. Schers H, Webster S, van den Hoogen H, Avery A, Grol R, van den Bosch W. Continuity of care in general practice: a survey of patients' views. Br J Gen Pract. 2002;52(479):459-62.
31. Sidaway-Lee K, Gray DP, Evans P. A method for measuring continuity of care in day-to-day general practice: a quantitative analysis of appointment data. Br J Gen Pract. 2019;69(682):e356-e62.
32. Baird B, Charles A, Honeyman M, Maguire D, Das P. Understanding pressures in general practice. The King's Fund. 2016.
33. Ministry of Health and Care Services. Handlingsplan for allmennlegetjenesten. Attraktiv, kvalitetssikker og teambasert 2020-2024. Oslo.2020 [Available from: https://www.regjeringen.no/contentassets/96f6581333ee48559cdabf23c8772294/handlingsplan-for-allmennleger.pdf.

For peer review only - http://bmjopen.bmj.com/site/about/guidelines.xhtml

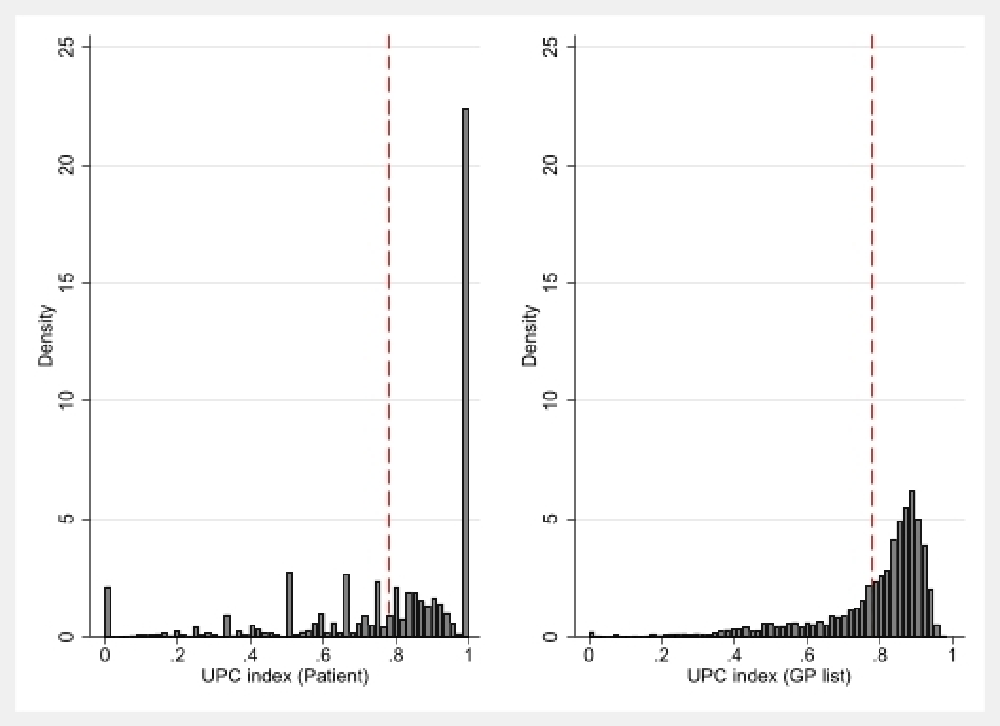

139x101mm (96 x 96 DPI)

Supplemenary file A

Supplementary file A shows the estimated association between the three outcomes and the UPC$^{GP\ list}$ continuity index, using a piecewise linear function for age, income, number of GP visits, and number of hospital admissions (linear splines). All models were estimated with three knots at the 25th, 50th and 75th percentile for the continues variables.

Appendix table 1. Linear splines

	(1) Emergency admission	(2) Emergency admission for ACSC	(3) Mortality
Index score	-0.025***	-0.006*	0.000
	(-3.43)	(-1.99)	(0.35)
N	757873	757873	757873
R^2	0.092	0.051	0.050

t statistics in parentheses. Standard errors clustered at GP level.
In all estimations, control variables include individual patient characteristics listed in table 1 and municipality-fixed effects.
* $p < 0.05$, ** $p < 0.01$, *** $p < 0.001$

For peer review only - http://bmjopen.bmj.com/site/about/guidelines.xhtml

The RECORD statement – checklist of items, extended from the STROBE statement, that should be reported in observational studies using routinely collected health data.

	Item No.	STROBE items	Location in manuscript where items are reported	RECORD items	Location in manuscript where items are reported (pages refer to "clean copy")
**Title and abstract**					
	1	(a) Indicate the study's design with a commonly used term in the title or the abstract (b) Provide in the abstract an informative and balanced summary of what was done and what was found		RECORD 1.1: The type of data used should be specified in the title or abstract. When possible, the name of the databases used should be included.<br><br>RECORD 1.2: If applicable, the geographic region and timeframe within which the study took place should be reported in the title or abstract.<br><br>RECORD 1.3: If linkage between databases was conducted for the study, this should be clearly stated in the title or abstract.	Title - page 1<br>Abstract<br>Page 2 line 7<br><br>Abstract<br>Page 2 line7<br><br>Abstract<br>Page 2 line 7
**Introduction**					
Background rationale	2	Explain the scientific background and rationale for the investigation being reported			Introduction page 4-6
Objectives	3	State specific objectives, including any prespecified hypotheses			Introduction<br>Page 6 line 7-12
**Methods**					
Study Design	4	Present key elements of study design early in the paper			Page 7 line7+
Setting	5	Describe the setting, locations, and relevant dates, including			Page 7 line7+

For peer review only

		periods of recruitment, exposure, follow-up, and data collection			
Participants	6	*(a) Cohort study* - Give the eligibility criteria, and the sources and methods of selection of participants. Describe methods of follow-up<br>*Case-control study* - Give the eligibility criteria, and the sources and methods of case ascertainment and control selection. Give the rationale for the choice of cases and controls<br>*Cross-sectional study* - Give the eligibility criteria, and the sources and methods of selection of participants<br><br>*(b) Cohort study* - For matched studies, give matching criteria and number of exposed and unexposed<br>*Case-control study* - For matched studies, give matching criteria and the number of controls per case		RECORD 6.1: The methods of study population selection (such as codes or algorithms used to identify subjects) should be listed in detail. If this is not possible, an explanation should be provided.<br><br>RECORD 6.2: Any validation studies of the codes or algorithms used to select the population should be referenced. If validation was conducted for this study and not published elsewhere, detailed methods and results should be provided.<br><br>RECORD 6.3: If the study involved linkage of databases, consider use of a flow diagram or other graphical display to demonstrate the data linkage process, including the number of individuals with linked data at each stage.	Page 8 line 1-14<br><br>Use complete population data, major selection is registered contacts, that is a valid variable in the national databases for health care use.<br><br>Page 7 line 8-22
Variables	7	Clearly define all outcomes, exposures, predictors, potential confounders, and effect modifiers. Give diagnostic criteria, if applicable.		RECORD 7.1: A complete list of codes and algorithms used to classify exposures, outcomes, confounders, and effect modifiers should be provided. If these cannot be reported, an explanation should be provided.	Page 8 line 15 to page 10 line 20
Data sources/ measurement	8	For each variable of interest, give sources of data and details of methods of assessment (measurement).			Page 8 line 15- page 10 line 20

For peer review only

		Describe comparability of assessment methods if there is more than one group			
Bias	9	Describe any efforts to address potential sources of bias			Page 11 line 2-16
Study size	10	Explain how the study size was arrived at			Page 8 line 1-14
Quantitative variables	11	Explain how quantitative variables were handled in the analyses. If applicable, describe which groupings were chosen, and why			Page 10line 22 - page 11 line 25
Statistical methods	12	(a) Describe all statistical methods, including those used to control for confounding<br>(b) Describe any methods used to examine subgroups and interactions<br>(c) Explain how missing data were addressed<br>(d) *Cohort study* - If applicable, explain how loss to follow-up was addressed<br>*Case-control study* - If applicable, explain how matching of cases and controls was addressed<br>*Cross-sectional study* - If applicable, describe analytical methods taking account of sampling strategy<br>(e) Describe any sensitivity analyses			Page 10 line 22 – page 12 line 14
Data access and cleaning methods		..		RECORD 12.1: Authors should describe the extent to which the investigators had access to the database population used to create the study population.	Page 25 line 7-8 (Use complete national register data as source Registries use

For peer review only

				RECORD 12.2: Authors should provide information on the data cleaning methods used in the study.	strict control on in-data.) See also page 7-8
Linkage		..		RECORD 12.3: State whether the study included person-level, institutional-level, or other data linkage across two or more databases. The methods of linkage and methods of linkage quality evaluation should be provided.	Linking described Page 7 line 8-22
**Results**					
Participants	13	(a) Report the numbers of individuals at each stage of the study (*e.g.*, numbers potentially eligible, examined for eligibility, confirmed eligible, included in the study, completing follow-up, and analysed)<br>(b) Give reasons for non-participation at each stage.<br>(c) Consider use of a flow diagram		RECORD 13.1: Describe in detail the selection of the persons included in the study (*i.e.,* study population selection) including filtering based on data quality, data availability and linkage. The selection of included persons can be described in the text and/or by means of the study flow diagram.	Page 8 line 1-14
Descriptive data	14	(a) Give characteristics of study participants (*e.g.*, demographic, clinical, social) and information on exposures and potential confounders<br>(b) Indicate the number of participants with missing data for each variable of interest<br>(c) *Cohort study* - summarise follow-up time (*e.g.*, average and total amount)			Table 1
Outcome data	15	*Cohort study* - Report numbers of outcome events or summary measures over time			Table 2

For peer review only

		Case-control study - Report numbers in each exposure category, or summary measures of exposure<br>*Cross-sectional study* - Report numbers of outcome events or summary measures			
Main results	16	(a) Give unadjusted estimates and, if applicable, confounder-adjusted estimates and their precision (e.g., 95% confidence interval). Make clear which confounders were adjusted for and why they were included<br>(b) Report category boundaries when continuous variables were categorized<br>(c) If relevant, consider translating estimates of relative risk into absolute risk for a meaningful time period			Table 2<br>Unadjusted estimates are not given, to be able to present different models in the same tables.
Other analyses	17	Report other analyses done—e.g., analyses of subgroups and interactions, and sensitivity analyses			Page 11 line22-23 and<br>page 11 line 17-19
**Discussion**					
Key results	18	Summarise key results with reference to study objectives			Page 18<br>line 5-15
Limitations	19	Discuss limitations of the study, taking into account sources of potential bias or imprecision. Discuss both direction and magnitude of any potential bias		RECORD 19.1: Discuss the implications of using data that were not created or collected to answer the specific research question(s). Include discussion of misclassification bias, unmeasured confounding, missing data, and changing eligibility over time, as they pertain to the study being reported.	Page 21 line 13<br>page 22 line 7

For peer review only

Interpretation	20	Give a cautious overall interpretation of results considering objectives, limitations, multiplicity of analyses, results from similar studies, and other relevant evidence			Page 91-21<br>And page 22-23
Generalisability	21	Discuss the generalisability (external validity) of the study results			Page 22 line 10-11
**Other Information**					
Funding	22	Give the source of funding and the role of the funders for the present study and, if applicable, for the original study on which the present article is based			Page 24 line 1
Accessibility of protocol, raw data, and programming code		..		RECORD 22.1: Authors should provide information on how to access any supplemental information such as the study protocol, raw data, or programming code.	Page 24 line 9

*Reference: Benchimol EI, Smeeth L, Guttmann A, Harron K, Moher D, Petersen I, Sørensen HT, von Elm E, Langan SM, the RECORD Working Committee. The REporting of studies Conducted using Observational Routinely-collected health Data (RECORD) Statement. *PLoS Medicine* 2015; in press.

*Checklist is protected under Creative Commons Attribution (CC BY) license.

For peer review only
